# Achieving robust somatic mutation detection with deep learning models derived from reference data sets of a cancer sample

**DOI:** 10.1186/s13059-021-02592-9

**Published:** 2022-01-07

**Authors:** Sayed Mohammad Ebrahim Sahraeian, Li Tai Fang, Konstantinos Karagiannis, Malcolm Moos, Sean Smith, Luis Santana-Quintero, Chunlin Xiao, Michael Colgan, Huixiao Hong, Marghoob Mohiyuddin, Wenming Xiao

**Affiliations:** 1 Roche Sequencing Solutions, Santa Clara, CA 95050 USA; 2 The Center for Biologics Evaluation and Research, U.S. Food and Drug Administration, 10903 New Hampshire Avenue, Silver Spring, MD 20993 USA; 3 National Center for Biotechnology Information, National Library of Medicine, National Institutes of Health, Bethesda, MD USA; 4 Office of Oncological Diseases, Office of New Drug, Center for Drug Evaluation and Research, U.S. Food and Drug Administration, 10903 New Hampshire Avenue, Silver Spring, MD 20993 USA; 5 Bioinformatics branch, Division of Bioinformatics and Biostatistics, National Center for Toxicological Research, U.S. Food and Drug Administration, 3900 NCTR Road, Jefferson, AR 72079 USA

**Keywords:** Somatic mutation, Deep learning, Convolutional neural networks, Well-characterized somatic reference samples, Model training strategies

## Abstract

**Background:**

Accurate detection of somatic mutations is challenging but critical in understanding cancer formation, progression, and treatment. We recently proposed NeuSomatic, the first deep convolutional neural network-based somatic mutation detection approach, and demonstrated performance advantages on in silico data.

**Results:**

In this study, we use the first comprehensive and well-characterized somatic reference data sets from the SEQC2 consortium to investigate best practices for using a deep learning framework in cancer mutation detection. Using the high-confidence somatic mutations established for a cancer cell line by the consortium, we identify the best strategy for building robust models on multiple data sets derived from samples representing real scenarios, for example, a model trained on a combination of real and spike-in mutations had the highest average performance.

**Conclusions:**

The strategy identified in our study achieved high robustness across multiple sequencing technologies for fresh and FFPE DNA input, varying tumor/normal purities, and different coverages, with significant superiority over conventional detection approaches in general, as well as in challenging situations such as low coverage, low variant allele frequency, DNA damage, and difficult genomic regions

**Supplementary Information:**

The online version contains supplementary material available at 10.1186/s13059-021-02592-9.

## Background

Somatic mutations are critical cancer drivers. Accurate somatic mutation detection enables precise diagnosis, prognosis, and treatment of cancer patients [1]. Recently, the Somatic Mutation Working Group in the FDA-led Sequencing Quality Control Phase II (SEQC2) consortium characterized matched tumor-normal reference samples: a human triple-negative breast cancer cell line (HCC1395) and a normal cell line derived from B lymphocytes (HCC1395BL) [2, 3], both from the same donor. Using orthogonal sequencing technologies, multiple sequencing replicates, and multiple bioinformatics analysis pipelines, the SEQC2 consortium has developed a well-defined reference call set of somatic single nucleotide variants (SNVs) and small insertion/deletions (INDELs) for HCC1395. In contrast to earlier efforts to establish benchmarking data for somatic mutation detection [4–7], this dataset has been well-characterized by the SEQC2 consortium and the truth set was defined with a comprehensive process yielding a high validation rate (>99.9%) [3] and used in benchmarking the performance of WGS and WES studies [2].

Derived from the first comprehensive and well-characterized paired tumor-normal reference cancer samples, this data set along with the accompanying sequencing data prepared at multiple sites with multiple technologies provides a unique resource for learning-based somatic mutation detection techniques.

Here, we performed an in-depth analysis of the contribution of SEQC2 data to deriving deep learning models that can achieve high robustness for detecting cancer mutations across real scenarios. We employed NeuSomatic [8], the first convolutional neural network (CNN)-based somatic mutation detection approach to identify effective model-building strategies and provide suggestions for training learning-based techniques. Unlike the conventional somatic mutation callers that use a set of hand-crafted sequencing features to distinguish somatic mutations from background noise, germline variants, and/or cross-contaminations through various statistical/algorithmic modeling methods [9–17], the network trained models can capture important mutation signals directly from read alignment and genomic context without manual intervention. This capability provides a framework that can easily be applied to different problem statements including diverse sequencing technologies, cancer types, tumor and normal purities, and mutation allele frequencies through training on real-world data. In addition, we applied these model-building strategies to train the random forest classifier implemented in Octopus [17], to show how well these strategies generalize to other machine learning-based approaches.

Our analysis of the SEQC2 reference data set demonstrates that the deep learning models can help to overcome the main challenges in somatic mutation detection which are not easy to resolve using conventional techniques. The models and strategies derived from our study can thus provide the research community with actionable best practices for robust cancer mutation detection.

## Results

### Reference samples and datasets

For full-spectrum analysis of the somatic mutation detection problem, we used the first comprehensively characterized whole-genome reference tumor-normal paired breast cancer cell lines (HCC1395 and HCC1395BL), developed by the Somatic Mutation Working Group of the SEQC2 consortium [2, 3]. We leveraged high-confidence somatic mutations (39,536 SNVs and 2020 INDELs) derived by the consortium as our ground truth set (Additional file 1: Fig. S1). For a broad assessment of consistency and reproducibility of predictions, we used a total of 119 replicates from diverse data sets representing realistic cancer detection applications including real whole-genome sequencing (WGS), whole exome sequencing (WES), and AmpliSeq targeted sequencing. The data sets had a wide range of coverages (10x to 2000x), tumor purities (5 to 100%), normal tissue purities (95% and 100%), and library preparations, using both formalin-fixed paraffin-embedded (FFPE) and fresh DNA, which was sequenced with multiple platforms at eight centers (see the “Methods” section).

### Analysis of different model-building strategies

We compared the robustness of different model-building strategies by training and evaluating NeuSomatic networks both in standalone mode (shown as NeuSomatic-S) and ensemble mode, where predictions reported by MuTect2 [9], MuSE [10], SomaticSniper [11], Strelka2 [12], and VarDict [13] were also included as input features in addition to the raw data.

As the baseline comparison network model, we used the model trained using in silico spike-ins from the DREAM Challenge Stage 3 data set [18]. Despite the large discrepancies between the sample types, sequencing platforms, coverages, spike-in mutation frequencies, and heterogeneity of the samples used to train the DREAM3 model, this model outperformed most of the other conventional techniques across the real cancer data sets with diverse characteristics (Additional file 1: Fig. S2). Although this superiority reflects robustness to the stated variations, it also suggests that through learning the sequencing features and mutation signatures from real cancer samples, predictions can be improved further, especially for INDELs and for the high-coverage (2000x) PCR-enriched AmpliSeq data set.

To identify the most effective strategies for building network models using the SEQC2 reference samples, we evaluated nine additional training approaches (see the “Methods” section) (Additional file 2: Table S1). The first model (SEQC-WGS-Spike) was trained on a set of in silico WGS tumor-normal replicates, generated by spiking mutations into distinct normal replicates. The second model (SEQC-WGS-GT-50) was trained using the ground truth somatic mutations in HCC1395 on a set of real WGS replicate pairs with 50% of the genome. The third model (SEQC-WGS-GT50-SpikeWGS10) was prepared by adding 10% of the training data from the first model to those for the second model to take advantage of both a high number of spike-in mutations and the realistic somatic mutations. These three models were tested on all data sets. For the specific data sets like FFPE and WES, we also prepared six additional specialized models using a set of synthetic pairs of in silico tumor and normal replicates. For all models, we evaluated the performance on the 50% held-out region of the genome (not used to construct the SEQC-WGS-GT-50 model).

**Fig. 1:**
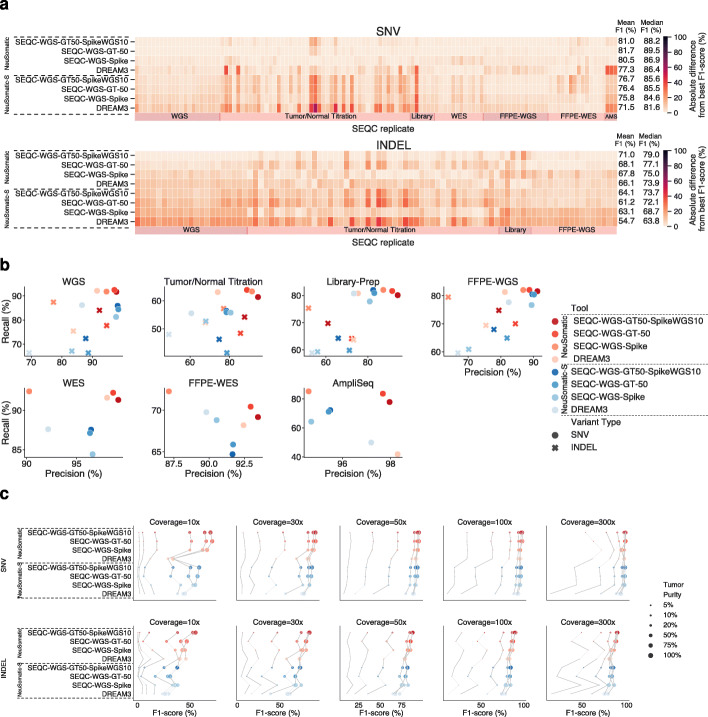
Overall performance of different NeuSomatic network trained models on 119 replicates from the SEQC2 data set. **a** The models trained on SEQC2 data achieved consistent superiority over the DREAM model across diverse sets of replicates of different purities/coverages in WGS, WES, FFPE, AmpliSeq (AMS), and different library preparation data sets. In this subfigure, for each replicate, the best F1-score was computed across different approaches. The heatmaps illustrate the absolute difference between the F1-score of any of the network models according to the best F1-score. In each panel, the mean F1-score is shown for each approach across 119 replicates. **b** Precision-recall analysis of different network models on different SEQC2 data sets. Each datapoint shows the average precision and recall values for SNVs and INDELs across the samples in a given dataset. **c** F1-score (%) comparison for different network models across different coverages (10×–300×) and tumor purities (5–100%). For a given coverage, results of different models on the same tumor purity are connected for better illustration.

Employing the models trained on SEQC2 samples, we boosted the average DREAM3 model performance by an additional mean F1-score improvement of ~4–5% (Fig. 1a, b; Additional file 1: Fig. S3-6). In general, the SEQC-WGS-GT50-SpikeWGS10 network model, trained on a mixture of real and spike-in mutations had higher average performance compared with other models across different samples and variant types. This may relate to the fact that it used both a large amount of spiked training data and a high number of realistic cancer mutations. The SEQC-WGS-GT-50 model, which was trained solely on real samples, performed slightly (~0.7%) better for SNVs, but it had ~3% lower F1-score for INDELs compared with the SEQC-WGS-GT50-SpikeWGS10 model. This may relate to the lower number of INDELs in the training data when only the real samples were used for training. The SEQC-WGS-Spike model, trained solely on spiked mutations, performed slightly worse than the two other models, which used real somatic mutations for training. This observation highlights the importance of real reference cancer samples in improving somatic mutation detection accuracy.

Performance comparison of different models across different tumor purities and sequencing coverages revealed that the models trained on the ground truth variants yielded the greatest advantage over other schemes (Fig. 1c), particularly for more challenging cases such as lower coverage (e.g., ~20% F1-score advantage for a sample with 10x coverage and 100% purity). The SEQC-WGS-Spike model, trained on in silico tumors, also showed lower precision compared with models trained on the reference call set for SEQC2 samples. In general, INDELs, low purity, and low coverage samples benefited the most from training on SEQC2 data.

Models trained on SEQC2 data consistently outperformed other models across different library preparation protocols and DNA input amounts (Additional file 1: Fig. S4).

The SEQC2 models trained on fresh samples consistently outperformed other models, with a more than 4% mean F1-score advantage, despite the presence of FFPE artifacts, and remained largely invariant to fixing time and the matched normal sample used, demonstrating robustness to FFPE processing (Additional file 1: Fig. S6). Training the network on FFPE samples seemed to improve only INDEL prediction.

To show the generalizability of these model-building strategies to other ML-based techniques, we used the same training datasets to train random forest classifiers for Octopus [17] (Additional file 1: Fig. S7-S11). Employing these models trained on SEQC2 samples, we achieved an average of ~10% mean F1-score improvement over Octopus-hard, which uses hard filtering to identify PASS calls. Similar to the models trained on NeuSomatic, the SEQC-WGS-GT50-SpikeWGS10 Octopus random forest model achieved the highest average accuracy among different random forest models with 0.7% and 4.1% mean F1-score improvement for SNVs and INDELs over the classifier trained on the DREAM3 dataset. Despite the advantage observed in training Octopus on SEQC2 data, NeuSomatic trained networks still outperformed Octopus.

Given the superiority of SEQC-WGS-GT50-SpikeWGS10, we used this model as the default NeuSomatic for comparison with other techniques. We compared the robustness of this model against eight widely used somatic mutation calling algorithms including MuTect2 [9], MuSE [10], SomaticSniper [11], Strelka2 [12], VarDict [13], Lancet [14], DRAGEN [16], and Octopus [17] with two different filtering strategies, one with hard filtering (Octopus-hard) and one with random forest filtering trained on SEQC-WGS-GT50-SpikeWGS10 data (Octopus-RF).

**Fig. 2:**
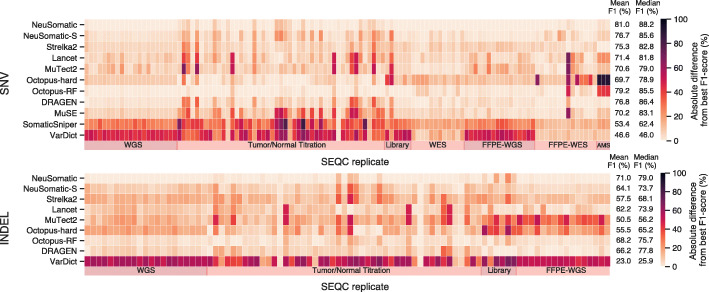
Overall performance of different somatic mutation callers on 119 replicates in the SEQC2 data set. The NeuSomatic model trained on SEQC2 data achieved consistent superiority over other techniques across diverse sets of replicates of different purities/coverages in WGS, WES, FFPE, AmpliSeq (AMS), and different library preparation data sets. For each replicate, the best F1-score was computed across different approaches. The heatmaps illustrate the absolute difference between the F1-score of any of the network models according to the best F1-score. In each panel, the mean F1-score is shown for each approach across 119 replicates. Here, the SEQC-WGS-GT50-SpikeWGS10 trained models were used for NeuSomatic and NeuSomatic-S

The proposed model-building strategy was consistently best across various sample types and sequencing strategies, outperforming other techniques by more than 1.8% and 2.8% in terms of the mean absolute F1-score for SNVs and INDELs, respectively. Similarly, we observed more than 1.8% and 1.2% median F1-score superiority over other techniques across all samples for SNVs and INDELs, respectively (Fig. 2; Additional file 1: Fig. S2). In general, the observed superiority in the F1-score resulted from both high precision and high recall (Additional file 1: Fig. S12).

### WGS dataset

**Fig. 3:**
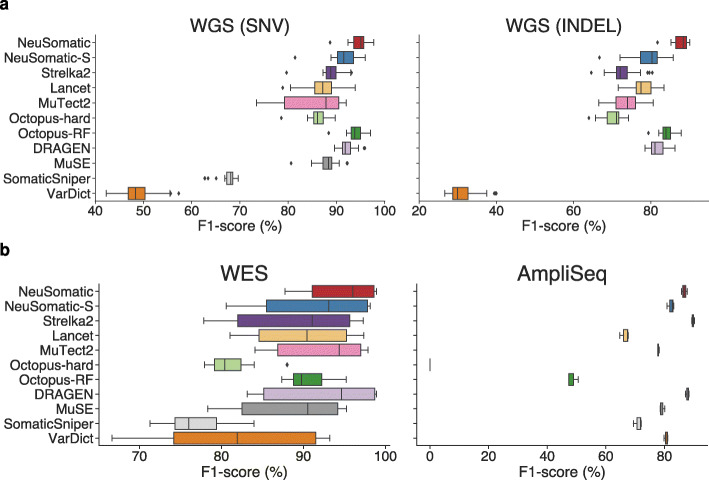
Performance comparison on WGS, WES, and AmpliSeq data sets. **a** F1-score (%) comparison on 21 WGS replicates for SNVs and INDELs. **b** SNV F1-score (%) comparison on WES and AmpliSeq data sets. Here, the SEQC-WGS-GT50-SpikeWGS10 trained models were used for NeuSomatic and NeuSomatic-S

To assess robustness to variations in sequencing centers and platforms, we investigated 21 WGS replicates sequenced in six sequencing centers using the HiSeq X Ten, HiSeq 4000, and NovaSeq S6000 platforms (Fig. 3a; Additional file 2: Table S2). With minor variations across replicates, the NeuSomatic SEQC-WGS-GT50-SpikeWGS10 model yielded average F1-scores of 94.6% and 87.9% for SNVs and INDELs with more than 0.6% and 3.7% superiority, respectively, over the mean F1-scores of other conventional schemes, demonstrating robustness and reproducibility (Fig. 3a).

### Tumor purity and contaminated normal

**Fig. 4:**
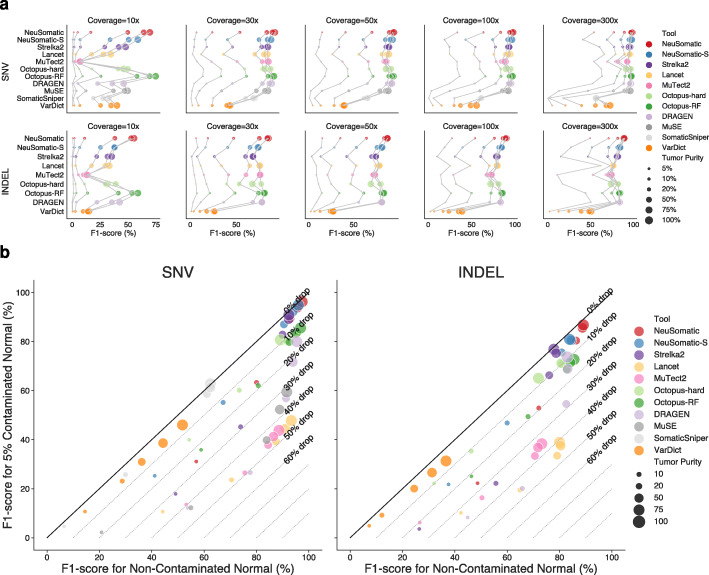
Performance comparisons on the tumor/normal titration dataset. **a** F1-score (%) comparison for different somatic mutation callers across different coverages (10x–300) and tumor purities (5–100%). For a given coverage, results of different callers on the same tumor purity are connected for better illustration. **b** Robustness to tumor contamination in the matched normal: F1-score (%) when matched normal was mixed with 5% of tumor is shown versus the F1-score (%) at pure normal for 80x coverage and 5–100% tumor purities. Here, the SEQC-WGS-GT50-SpikeWGS10 trained models were used for NeuSomatic and NeuSomatic-S

To investigate performance variations across different tumor purities, we mixed samples of tumor HCC1395 gDNA with normal HCC1395BL gDNA at different ratios representing 5–100% tumor purity levels. For each of these tumor purity levels, we evaluated the performance at 10x–300x sequencing coverages (Fig. 4; Additional file 1: Fig. S13; Additional file 2: Table S3). In general, for SNVs, NeuSomatic and Octopus-RF performed better than other techniques, while for INDELs NeuSomatic outperformed other approaches across the tumor purity and coverage variations.

We further analyzed the robustness to 5% tumor contamination in the normal sample for tumor sample purities ranging from 10 to 100% at 80x coverage (Fig. 4b). We observed high robustness of the NeuSomatic model trained on the SEQC2 data to tumor-normal cross-contamination with less than 5% median absolute change in F1-score. Among the other techniques with a high F1-score at pure normal, Strelka2 also showed high robustness to tumor contamination (8.4% median change in F1-score). MuTect2, MuSE, and Lancet, despite having high F1-scores for the pure normal scenario, experienced significant drops of up to ~50% in F1-score when contaminated normal data were used.

### Library preparation and DNA input

**Fig. 5:**
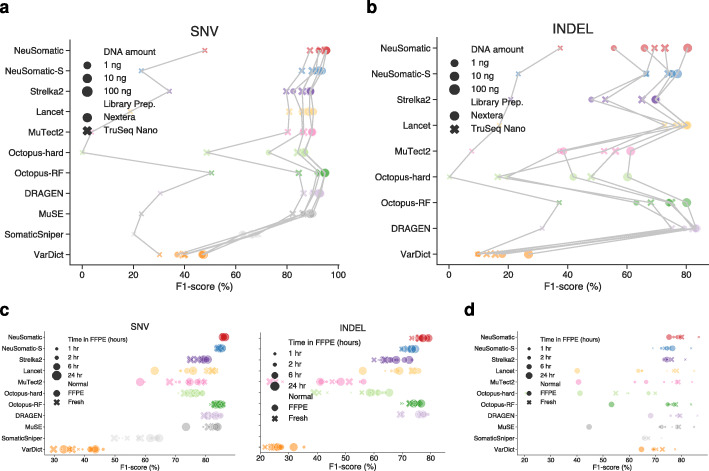
Performance comparisons on the library preparation and FFPE data sets. **a**, **b** F1-score (%) comparison for different somatic mutation callers across different library kits and DNA amounts. Results of different callers for the same library preparation kit and DNA amount are connected for better illustration. **c** F1-score (%) comparison for different somatic mutation callers on 16 FFPE WGS replicates with FFPE or fresh matched normal. **d** SNV F1-score (%) comparison for different somatic mutation callers on 14 FFPE WES replicates with FFPE or fresh matched normal. Here, the SEQC-WGS-GT50-SpikeWGS10 trained models were used for NeuSomatic and NeuSomatic-S

To measure the effect of library preparation on prediction robustness, we used our models to test the six replicates prepared using TruSeq-Nano and Nextera Flex protocols across three DNA input amounts: 1ng, 10ng, and 100ng. The NeuSomatic model trained on the SEQC2 data consistently outperformed other techniques across different library preparation protocols and DNA input amounts (Fig. 5a, b; Additional file 2: Table S4). For the 1 ng TruSeq-Nano libraries, all the methods had poor performance due to the limited effective coverage after removal of redundant reads (~7x). NeuSomatic models yielded ~0.6% mean F1-score improvement for SNVs over the best alternative, Octopus-RF. For INDELs, DRAGEN performed the best followed by Lancet, Octopus-RF, and NeuSomatic.

### Captured (WES) and targeted (AmpliSeq) panels

Evaluation of 12 WES replicates sequenced at six sequencing sites, as well as three replicates of AmpliSeq data, revealed that although the models trained on SEQC2 WGS samples had different coverage and platform biases, they performed equally well on targeted sequencing data with up to 2000x coverage (Fig. 3b; Additional file 2: Table S5 and S6)**.** On the WES data set, models trained on WES and WGS data performed similarly with ~95% mean F1-score, achieving more than 2.4% improvement over alternative schemes. On the AmpliSeq data set, DRAGEN performed the best, followed by Strelka2 and the NeuSomatic SEQC2 model, which achieved more than 10% higher F1-score than other schemes.

### Effect of FFPE processing

To measure the robustness of prediction on FFPE processed samples, we used data from eight WGS and seven WES FFPE replicates sequenced at two centers and prepared with different formaldehyde fixation times (1, 2, 6, and 24 h), matched with either FFPE or fresh normal samples (more information at [2]) (Fig. 5c, d; Additional file 2: Table S7 and S8). The NeuSomatic SEQC2 models trained on fresh samples consistently outperformed other techniques, with ~2% mean F1-score advantage for SNVs and INDELs despite the presence of FFPE artifacts and remained largely invariant to fixing time and the matched normal sample used, demonstrating broad robustness to FFPE processing.

### Sample-specific models

**Fig. 6:**
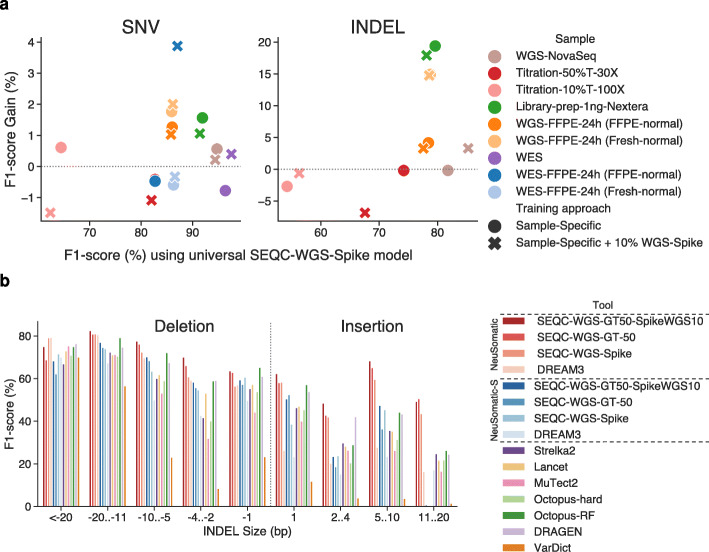
Performance analysis of the sample-specific models, and comparisons on different INDEL sizes. **a** Effect of training on the target sample for nine replicates from different SEQC2 data sets. For each sample, two sample-specific models were used: one was trained on only the target sample, and the other with an additional 10% of the training data from the SEQC-WGS-Spike model. **b** Average performance of somatic mutation callers for different INDEL sizes on SEQC2 replicates. Negative INDEL sizes reflect deletions

Having found that the models trained on all the SEQC2 data consistently performed better than other somatic mutation detection schemes, we also explored whether using sample-specific data to train models could give us an additional accuracy boost. For nine replicate pairs across different SEQC2 data sets, we trained models using the in silico tumor-normal replicates prepared for that sample. On average, the sample-specific models yielded ~0.5% SNV and ~5% INDEL F1-score improvements over the universal SEQC-WGS-Spike model (Fig. 6a; Additional file 2: Table S9). The library-prep and FFPE samples benefited the most from sample-specific training, with more than 1.6% and 14.8% F1-score improvement.

### INDEL performance

Assessing the INDEL predictions revealed that although the NeuSomatic models did not use local assembly explicitly, they still consistently surpassed other approaches including the assembly-based techniques like Lancet and Octopus across a broad range of INDEL size, coverage, and tumor purity (Fig. 6b; Additional file 1: Fig. S14 and S15). Overall, for insertions and deletions of more than one base pair, the NeuSomatic SEQC2 trained models yielded 18.7% and 5% superiority in F1-score over DRAGEN and Octopus-RF, the best alternative techniques.

### Performance analysis for different variant allele frequencies (VAFs)

**Fig. 7:**
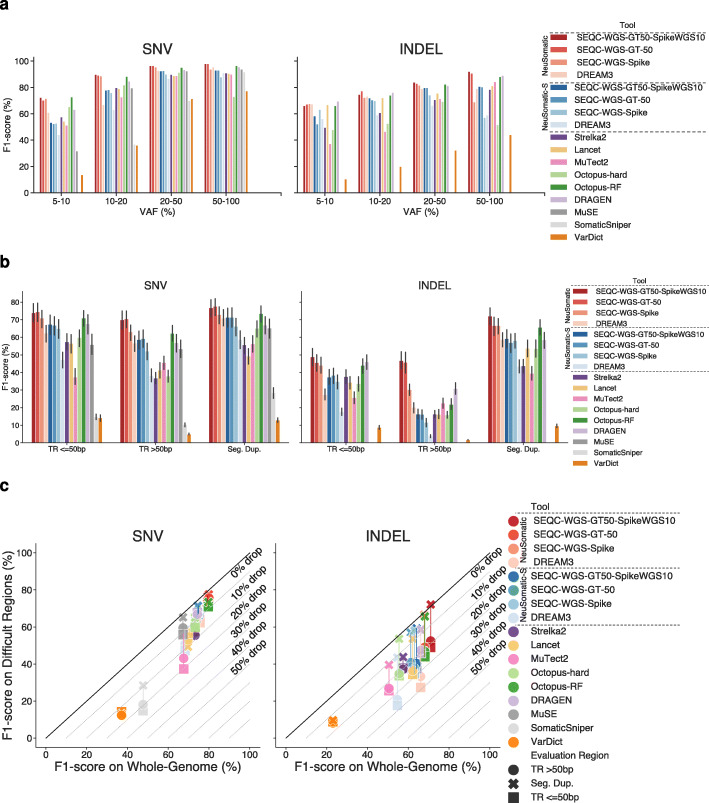
Performance comparisons on different VAF distributions and difficult regions. **a** Performance analysis of different somatic callers on different VAF ranges across all replicates in the SEQC2 data sets. **b** Performance analysis of different somatic callers on difficult regions including tandem repeats (TR) of different sizes and segmental duplications. **c** Comparison of the F1-score (%) on the whole genome against the F1-score (%) on difficult genomic regions including tandem repeats (TR) of different sizes and segmental duplications

Analyzing the prediction accuracies for different variant allele frequency (VAF) ranges revealed higher robustness of the proposed training strategies to VAF variation, in a broad range of 5–100%, with a larger advantage for VAFs with 5–20% (Fig. 7a; Additional file 1: Fig. S16-S18). The SEQC2 trained models clearly performed better than the DREAM3 model for mutations with 5–20% VAFs.

### Performance on difficult genomic regions

Performance comparison on error-prone genomic regions, including tandem repeats (TR) and segmental duplications, revealed that many other schemes experienced a significant drop of up to 40% in their prediction accuracies in such difficult-to-call regions (Fig. 7b, c; Additional file 1: Fig. S19 and S20). However, the models trained on SEQC2 data remained highly robust to genomic context complexity, with more than 6% mean F1-score superiority for both SNVs and INDELs over alternative schemes.

### Performance on non-SEQC2 samples

To evaluate the performance of the SEQC2 trained models on orthogonal samples, we employed the synthetic samples developed in [17], where two synthetic tumors were generated by spiking real pancreatic cancer (PACA) and breast cancer (BRCA) mutations into normal samples from Genome in a Bottle [19]’s NA24631 and NA12878 high-coverage Illumina data, respectively. The NA24631.PACA synthetic tumor has 60,110 mutations with 0.5–50% VAF and the NA12878.BRCA synthetic tumor has 5895 mutations with 1–20% VAF. The 265× NA24631.PACA tumor is paired with a 90x NA24631 normal and the 220x NA12878.BRCA tumor is paired with a 75x NA24631 normal.

Evaluating different somatic mutation callers on these two orthogonal samples revealed that DRAGEN and the NeuSomatic model trained on the SEQC2 data achieved higher accuracy compared with other approaches (Additional file 1: Fig. S22).

## Discussion

We explored the robustness of the deep learning models trained on the SEQC2 reference data sets in detecting somatic mutations across a diverse set of experimental settings seen with real cancer samples. These models, trained on WGS data sets from multiple platforms and centers, both using spike-in mutations and the set of ground truth somatic mutations derived from the SEQC2 studies, boosted somatic mutation detection performance to achieve the highest accuracy. This analysis highlighted model-building best practice strategies useful in various scenarios, i.e., a model trained on a combination of real and spike-in mutations achieved the highest average performance. Compared with the baseline DREAM3 network models, the proposed training strategy derived from SEQC2 reference data sets reduced false positives and false negatives for both SNVs and INDELs, improved insertion calling accuracies, and enhanced somatic mutation detection in difficult genomic regions by learning correct mutation signals. The better performance of NeuSomatic trained on mixtures of mutations was validated by two independent data sets (Additional file 1: Fig. S22). This strategy of model training can also be applied to other machine learning tools, as demonstrated for Octopus (Additional file 1: Fig. S7-S11).

While the SEQC2 trained models remained robust to tumor contamination in the matched normal data, many somatic calling approaches such as MuTect2, Lancet, and MuSE were degraded severely, because they rejected true somatic mutations that had corresponding reads in the normal dataset. Thus, their recall rate dropped significantly. The SEQC2 trained models proposed in this study, when trained on WGS data, performed equally well on targeted sequencing data. On the other hand, because Lancet uses a localized colored de Bruijn graph approach designed specifically for genome-wide somatic mutation detection, it is not suitable for targeted sequencing which typically covers less than 1 MB of the genome.

Analyzing prediction accuracies across variations in mutation type, INDEL length, VAF, and genomic region revealed that the proposed network models yielded the largest improvements over other schemes in challenging situations such as low coverage, less than 20% VAF, difficult genomic regions, INDELS longer than one base pair, FFPE with DNA damage, or contaminated matched normal. As a result of random sequencing errors and/or sample-specific artifacts with the true somatic mutations, conventional schemes had many false positives (and thus lower precision) that were often seen in low-complexity genomic regions (Fig. 7b, c; Additional file 1: Fig. S19 and S20). However, by training on WGS samples from multiple platforms and multiple centers, the network learned error-prone genomic contexts and thus consistently enhanced accuracy across different conditions including difficult-to-call low-complexity genomic contexts. Similarly, analysis of calls missed by alternative approaches such as Strelka2 also revealed that most of the private false-negative calls (see the “Methods” section for definition), which were predicted correctly by the SEQC2 models, had low VAF (Additional file 1: Fig. S21). This confirmed the capability of these models to accurately distinguish challenging low VAF calls from artifacts by learning the correct signature from raw data. Therefore, we suggest a multi-center, multi-platform approach that uses a combination of spike-in mutations as well as ground truth real somatic mutations as a best practice when developing or evaluating learning-based models for somatic variant detection.

## Conclusion

The cancer research community can benefit from the strategies developed in this study by using the NeuSomatic trained models we derived and following the methods proposed to extend the training to derive their dataset/sample-specific models and achieve the highest accuracy. Application of convolutional neural networks to detection of somatic sequence variants offers substantial improvements over existing alternatives, particularly in robustness to formalin fixation and to difficult genomic regions over a wide variety of conditions represented by the SEQC2 dataset.

## Methods

### SEQC2 tumor-normal sequencing data and ground truth

We used the SEQC2 reference matched samples, a human triple-negative breast cancer cell line (HCC1395) and a matched B lymphocyte-derived normal cell line (HCC1395BL), both derived from the same donor, in our analysis. Detailed sample information can be found in the SEQC2 reference data and call set manuscripts [2, 3]. The SEQC2 somatic mutation working group has established a reference call set of somatic mutations for these samples [3] (Additional file 1: Fig. S1). The call set was defined using multiple tumor-normal sequencing replicates from different sequencing centers, and orthogonal mutation detection bioinformatics pipelines. To assign confidence scores to the mutation calls and minimize platform, center, and pipeline specific biases, the SomaticSeq [20] machine learning framework was implemented to train a set of classifiers on in silico tumor-normal replicate pairs generated by spiking synthetic mutations into an HCC1395BL alignment file, matched with another HCC1395BL for each replicate pair. Using these classifiers, mutation calls were categorized into four confidence levels (HighConf, MedConf, LowConf, and Unclassified) based on cross-aligner and cross-sequencing-center reproducibility. HighConf and MedConf calls were grouped together as the “reference call set” of somatic mutations (v1.0), which contains a total of 39,536 SNVs and 2020 INDELs. The truth set for somatic mutations in HCC1395 is available to the community on NCBI’s ftp site (ftp://ftp-trace.ncbi.nlm.nih.gov/seqc/ftp/Somatic_Mutation_WG/).

All sequencing data used in this study are publicly available through NCBI’s SRA database (SRP162370). For all samples, the FastQ files were first trimmed using Trimmomatic [21] and then aligned with BWA-MEM (v0.7.15) [22] followed by Picard MarkDuplicates (https://broadinstitute.github.io/picard).

### Training data sets and models

Different training data sets were used to derive effective NeuSomatic CNN models using both in silico tumor replicates, prepared with synthetic spike-in mutations, and real tumor replicates with a known high-confidence truth set of somatic mutations (Additional file 2: Table S1).

#### DREAM3 model

As the baseline WGS model, we employed the DREAM3 model, developed recently [8] by training on ICGC-TCGA DREAM Challenge Stage 3 data [18]. The Stage 3 data set consists of a normal sample and a tumor sample constructed by computationally spiking 7903 SNVs and 7604 INDEL mutations into a healthy genome of the same normal sample with three different VAFs of 50%, 33%, and 20% to create synthetic but realistic tumor-normal pairs. An additional ~95K SNVs and ~95K INDELs were spiked into the tumor sample using BAMSurgeon [18] with similar VAF distributions to the original DREAM data for better network training. This model was trained by combining training data (in 50% of the genome) from five different tumor-normal purity settings of 100T:100N, 50T:100N, 70T:95N, 50T:95N, and 25T:95N [8]. The network was trained on ~29.2M candidate mutations detected (by scanning the alignment) in these five replicate pairs, which includes ~450K candidates with somatic SNV/INDEL labels and ~28.7M candidates, labeled as non-somatic.

#### TCGA model

As the baseline WES model, we used the TCGA model developed recently [8] by training on a set of 12 TCGA samples [23]. The tumor and normal alignments for each of these samples were mixed and split into two alignments of equal size. One alignment set was treated as the normal replicate and the other set was used to spike in mutations to construct the corresponding tumor replicate. For each sample, ~88K SNVs and ~44K INDELs were spiked in to generate a synthetic tumor sample for training. The network was trained on ~5.9M candidate mutations detected (by scanning the alignment) in these 12 replicate pairs, which included ~1.5M candidates with somatic SNV/INDEL labels and ~4.4M candidates, labeled as non-somatic.

#### SEQC-WGS-Spike model

To prepare the training data used to build this model, we employed BAMSurgeon to construct a synthetic tumor by spiking in silico mutations into one of the HCC1395BL replicates and pairing that with distinct HCC1395BL replicates as a normal match. Using this approach, we prepared ten in silico tumor-normal pairs. Eight of the ten replicate pairs were selected from WGS replicates sequenced at four different sites with 40x–95x mean coverage. The other two replicate pairs were created by merging multiple NovaSeq sequencing replicates from Illumina to obtain in silico ~220x tumor sample coverage and ~170x normal matched sample coverage. In each in silico tumor, we spiked in ~92K SNVs and ~22K INDELs. The VAFs of the spike-in mutations were randomly selected from a beta distribution (with shape parameters 휶 = 2 and 휷 = 5). For each of the ten replicate pairs, we also constructed an impure normal by mixing 95% normal and 5% tumor reads; in total, we used 20 in silico tumor-normal pairs to train the SEQC-WGS-Spike model. We then trained the network on ~272M candidate mutations detected (by scanning the alignment) in these 20 replicate pairs, which included ~2M candidates with somatic SNV/INDEL labels and ~270M candidates, labeled as non-somatic.

#### SEQC-WGS-GT-50 model

This model was constructed using the real WGS tumor-normal replicates accompanied by the SEQC2 HighConf truth set as the ground truth somatic mutations. We used eight WGS tumor-normal replicates as the base samples to train this model. The first seven WGS replicate pairs were from six different sequencing centers on HiSeq and NovaSeq platforms with mean coverage of 40x–95x, and the last one was constructed by combining nine NovaSeq sequencing replicates from Illumina to get a replicate pair with ~390x coverage. For each of these eight replicate pairs, we constructed two other purity variations, one with 95% normal purity by mixing 95% normal and 5% tumor reads and the other with 10% tumor purity by mixing 10% tumor and 90% normal reads. Thus, for each of the replicate pairs, we had a version with 100% pure tumor and normal, a version with 100% pure tumor matched with 95% pure normal, and a version with 10% pure tumor matched with 100% pure normal; in total, we used 24 tumor-normal replicates to train the SEQC-WGS-GT-50 model. To have unbiased evaluation, we only used 50% of the SEQC2 high-confidence regions of the genome to train this model and held out the other 50% for evaluation. To prepare the training and evaluation regions, we split the SEQC2 high-confidence regions of the genome into small regions of ~92K bps and randomly selected half of the fractured regions for training and the other half for evaluation. The training region excluded a 5-base pair segment around each of the gray-zone mutations, which contained LowConf-labeled calls and Unclassified-labeled calls with greater than 30% VAF. We then trained the network on ~137M candidate mutations detected (by scanning the alignment) in these 24 replicate pairs including ~416K candidates with somatic SNV/INDEL labels and ~136.5M candidates, labeled as non-somatic.

#### SEQC-WGS-GT50-SpikeWGS10 model

The training data for this model were prepared by combining 10% of the training candidates used for the SEQC-WGS-Spike model and all candidates from SEQC-WGS-GT-50. This combination took advantage of both a high number of spike-in mutations and the authentic somatic mutation characteristics seen in real tumor-normal data. We trained the network on the combined set of 164M candidate mutations, including ~574K candidates with somatic SNV/INDEL labels and ~163.5M candidates, labeled as non-somatic.

#### SEQC-WES-Spike model

Similar to the SEQC-WGS-Spike model, we used BAMSurgeon to construct seven in silico tumor-normal WES replicates to train this model. The replicate pairs were selected from the WES data set sequenced at four different sites with 60x–550x mean coverage. In each in silico tumor, we spiked in ~97K SNVs and ~19K INDELs. The VAFs of the spike-in mutations were randomly selected from a beta distribution (with shape parameters 휶 = 2 and 휷 = 5). We then trained NeuSomatic and NeuSomatic-S on ~3.7M candidate mutations detected (by scanning the alignment) in these seven replicate pairs, which included ~755K candidates with somatic SNV/INDEL labels and ~3M candidates, labeled as non-somatic.

#### SEQC-FFPE-Spike model

Similar to the SEQC-WGS-Spike model, we used BAMSurgeon to construct eight in silico tumor-normal WGS FFPE replicates to train this model. The replicate pairs were selected from the FFPE data set sequenced at two different sites with four different preparation times. In each in silico tumor, we spiked in ~92K SNVs and ~22K INDELs. The VAFs of the spike-in mutations were randomly selected from a beta distribution (with shape parameters *α* = 2 and *β* = 5). We also matched each in silico tumor with a fresh WGS replicate to include FFPE tumor versus fresh normal scenarios in our training. So, in total, we used 16 in silico tumor-normal pairs to train the SEQC-FFPE-Spike model. We trained NeuSomatic and NeuSomatic-S on ~191M candidate mutations detected (by scanning the alignment) in these eight replicate pairs, which included ~1.7M candidates with somatic SNV/INDEL labels and ~190M candidates, labeled as non-somatic.

#### SEQC-FFPE-WES-Spike model

Similar to other spike-in models, we used BAMSurgeon to construct seven in silico tumor-normal WES FFPE replicates to train this model. The replicate pairs were selected from the WES FFPE data set sequenced in two different sites and prepared across four varying time intervals. Because we do not have two normal replicates with the same FFPE preparation time and sequencing site for this data set, we mixed the tumor and normal alignments for each replicate pair and split the mixture into two alignments with equal size. We then treated one alignment as the normal replicate and spike-in mutations in the other to construct the tumor replicate. In each in silico tumor, we spiked in ~90K SNVs and ~19K INDELs. The VAFs of the spike-in mutations were randomly selected from a beta distribution (with shape parameters *α* = 2 and *β* = 5). We also matched each in silico tumor with a fresh WES replicate to include FFPE tumor versus fresh normal scenarios in our training; in total, we used 14 in silico tumor-normal pairs to train the SEQC-FFPE-WES-Spike model. We trained NeuSomatic and NeuSomatic-S on ~9.6M candidate mutations detected (by scanning the alignment) in these seven replicate pairs, which included ~1.4M candidates with somatic SNV/INDEL labels and ~8.2M candidates, labeled as non-somatic.

#### SEQC-WES-SpikeWGS10, SEQC-FFPE-SpikeWGS10, and SEQC-FFPE-WES-SpikeWGS10 models

The training data for these models were prepared by combining 10% of training candidates used for the SEQC-WGS-Spike model and respectively all candidates from SEQC-WES-Spike, SEQC-FFPE-Spike, and SEQC-FFPE-WES-Spike. This combination took advantage of both the high number of spike-in WGS mutations and the sample biases for WES and FFPE samples.

#### Sample-specific models

In addition to the above general-purpose models, we derived sample-specific models for a set of nine samples across multiple data types. The selected samples included a WGS sample sequenced by a NovaSeq instrument, a WES sample, a sample prepared with a Nextera Flex library-prep kit with 1ng DNA, a 30x WGS sample with 50% tumor purity, a 100x WGS sample with 10% tumor purity, and two WGS and two WES FFPE samples each treated with formalin for 24h and matched with fresh/FFPE normal samples. For each sample, we prepared the in silico tumor using random spikes. For the 10% tumor sample, the VAFs of the spike-in mutations were randomly selected from a beta distribution (with shape parameters *α* = 2 and *β* = 18). For the other samples, a beta distribution (with shape parameters *α* = 2 and *β* = 5) was used to select the VAFs. For each sample, we then trained the network models using the in silico tumor-normal replicates. In addition, for each sample, we trained a distinct model by combining 10% of training candidates used for the SEQC-WGS-Spike model and the training data derived for that sample.

### Somatic mutation detection algorithms

In addition to NeuSomatic, we used six somatic mutation callers, MuTect2 (4.beta.6) [9], SomaticSniper (1.0.5.0) [11], Lancet (1.0.7) [14] , Strelka2 (2.8.4) [12], MuSE (v1.0rc) [10], VarDict (v1.5.1) [13], DRAGEN (v3.7.5) [16], and Octopus (v0.7.4) [17], and ran each of them using the default parameters or parameters recommended by the user’s manual. For SomaticSniper, we used “-q 1 -Q 15 -s 1e-05.” For Lancet, we used “-cov-thr 10 -cov-ratio 0.005 -max-indel-len 50 -e 0.005.” The high-confidence outputs flagged as “PASS” in the resulting VCF files were applied to our comparison analysis. For VarDict, we also restricted calls to those with “Somatic” status. Results from each caller used for comparison were all mutation candidates that users would otherwise consider as “real” mutations detected by this caller. For Octopus, we ran in two different filtering modes of hard filtering (Octopus-hard) and filtering based on the pretrained random forest model (Octopus-RF). We then used Octopus “PASS” calls tagged with “SOMATIC” status.

For DRAGEN, we initially generated the index for the reference sequences using the parameters “--build-hash-table true --enable-cnv true --ht-alt-aware-validate false --ht-cost-coeff-seed-freq 0.5 --ht-cost-coeff-seed-len 1 --ht-cost-penalty-incr 0.7 --ht-cost-penalty 0 --ht-max-seed-freq 16 --ht-ref-seed-interval 1 --ht-seed-len 21 --ht-target-seed-freq 4.” For all the datasets with paired normal samples, we generated BED files to filter out systematic noise according to the DRAGEN v3.7 manual. For this step, each group of normal samples was processed separately. For example, each formalin-fixed paraffin-embedded WGS normal sample was processed with the DRAGEN’s somatic tumor-only pipeline using the parameters “--enable-variant-caller true --enable-map-align false --vc-detect-systematic-noise true.” All the resulting hard-filtered vcf files were provided as input to DRAGEN’s systematic noise BED generation pipeline with the parameters “--vc-systematic-noise-use-germline-tag true --vc-systematic-noise-method max.” Of note, for the WES samples, the “--vc-systematic-noise-method” parameter was set to “aggregate.” The computations wherein the tumor samples were paired with normal samples from different technologies, depth of coverage, or preparation (e.g., the FFPE WGS tumor samples paired with fresh WGS normal samples) omitted the systematic noise step. Similarly, the step was skipped in those computations where the cardinality of the normal samples set was less than three. The parameters used in all DRAGEN somatic tumor-normal computations were the following: “--enable-variant-caller true --enable-map-align false.” Finally, the systematic noise removal step, when applicable, was invoked with the parameter “--vc-systematic-noise <systematic_noise.bed>.”

We used NeuSomatic (0.1.4) in both ensemble and standalone mode. For ensemble mode, in addition to the candidate variants identified by the “alignment scanning” step of NeuSomatic, we also incorporated calls from five individual callers (MuTect2, SomaticSniper, Strelka2, MuSE, and VarDict) and represented their occurrence as additional input channels for each candidate variant. For NeuSomatic and NeuSomatic-S, we used “-scan_maf 0.01 -min_mapq 10 -snp_min_af 0.03 -snp_min_bq 15 -snp_min_ao 3 -ins_min_af 0.02 -del_min_af 0.02 -num_threads 28 -scan_window_size 500 -max_dp 100000” during preprocessing step. For training, we used “-coverage_thr 300 -batch_size 8000.”

### Difficult genomic regions

We used a set of difficult genomic regions derived by the Genome-in-a-Bottle consortium [24]. These regions included tandem repeats, in two different categories of less than 50bp and larger than 50bp, and segmental duplication regions. We evaluated different techniques on these regions to illustrate robustness to genomic context complexity.

### Analysis of false-negative calls

To better understand the difference in the performance of different techniques, we performed a set of pairwise comparisons between the VAF distribution of the false-negative (FN) SNVs of NeuSomatic against other schemes using the WGS data set (Additional file 1: Fig. S21). In each X-vs-Y analysis, we identified X_FN_, the set of ground truth SNVs missed by algorithm X (false negative in X) in at least 11 out of 21 WGS replicates. Similarly, we identified Y_FN_, the set of ground truth SNVs which were missed by algorithm Y (false negative in Y) in at least 11 out of 21 WGS replicates. We then computed the private false negatives of X, defined as X_FN_/Y_FN_, and the private false negatives of Y, defined as Y_FN_/X_FN_. For somatic mutations in each of those sets, we then computed the distribution of VAF.

### Evaluation process

For a fair comparison, we evaluated all the models and somatic mutation algorithms on the 50% hold-out genomic region which was not used for training the SEQC-WGS-GT-50 model. This ~1.4GB region contained ~21K SNVs and ~1.3K INDELs from the SEQC2 truth set for HCC1395 which were not used for training.

Calls labeled as HighConf and MedConf by the SEQC2 consortium for HCC1395 were grouped together as the “truth set” of somatic mutations used herein. We employed this truth set to compute the true positives and false negatives across all replicates of HCC1395 for all pipelines. For both SNVs and INDELs, a variant call is only assumed to be correct if it exactly matches a ground truth variant. As recommended by the SEQC2 consortium, we also blacklisted LowConf calls with low validation rates. Since this truth set had a VAF detection limit of 5% and depth detection limit of 50x, for calls at higher coverages or lower VAFs, we cannot ascertain their real somatic status. Thus, for private calls reported by any pipeline that was not in the truth set, we excluded calls which were deemed to be ambiguous from evaluation. In summary, for a tumor replicate that had a mean coverage of *C* and a tumor purity of *P*, if a pipeline had reported a private somatic mutation for this replicate (which was not in the truth set) that has *d* number of supporting reads, we only labeled this call as false positive if its projected number of supporting reads at 100% purity and 50x coverage was ≥3. In other words, this call was false positive if *d* ≥ 3*CP*/50; otherwise, we excluded this call from the evaluation.

Since the number of true indels in the evaluation region was very limited, we only reported SNV evaluations for WES and AmpliSeq data.

### Computational efficiency

We used the synthetic tumor-normal sample NA12878.BRCA with 220x tumor coverage and 75x normal coverage to compare the computational complexity of different somatic mutation callers (Additional file 2: Table S10).

## Supplementary Information

**Additional file 1:.** Supplementary Figures.

**Additional file 2:.** Supplementary Tables.

**Additional file 3:.** Review history

## Data Availability

All sequencing data used in this study are publicly available through NCBI’s SRA database (SRP162370) [25]. The bam files used to train NeuSomatic models are available on NCBI’s ftp site (https://ftp-trace.ncbi.nlm.nih.gov/ReferenceSamples/seqc/Somatic_Mutation_WG/data/DeepLearning_bams/). The code to create models, figures, and tables is deposited on zenodo under a Creative Commons Attribution 4.0 license [26].
