# Supplementary material for: Achieving robust somatic mutation detection with deep learning models derived from reference data sets of a cancer sample
Source: Genome Biol. 2022 Jan 7;23:12. doi: 10.1186/s13059-021-02592-9 (PMC8740374; doi:10.1186/s13059-021-02592-9)
Supplement: Supplementary file 1 — Additional file 1:. Supplementary Figures. [file 13059_2021_2592_MOESM1_ESM.pdf]

Sahraeian et al. Achieving robust somatic mutation detection with deep learning models derived from reference data sets of a cancer sample

Supplementary Figures

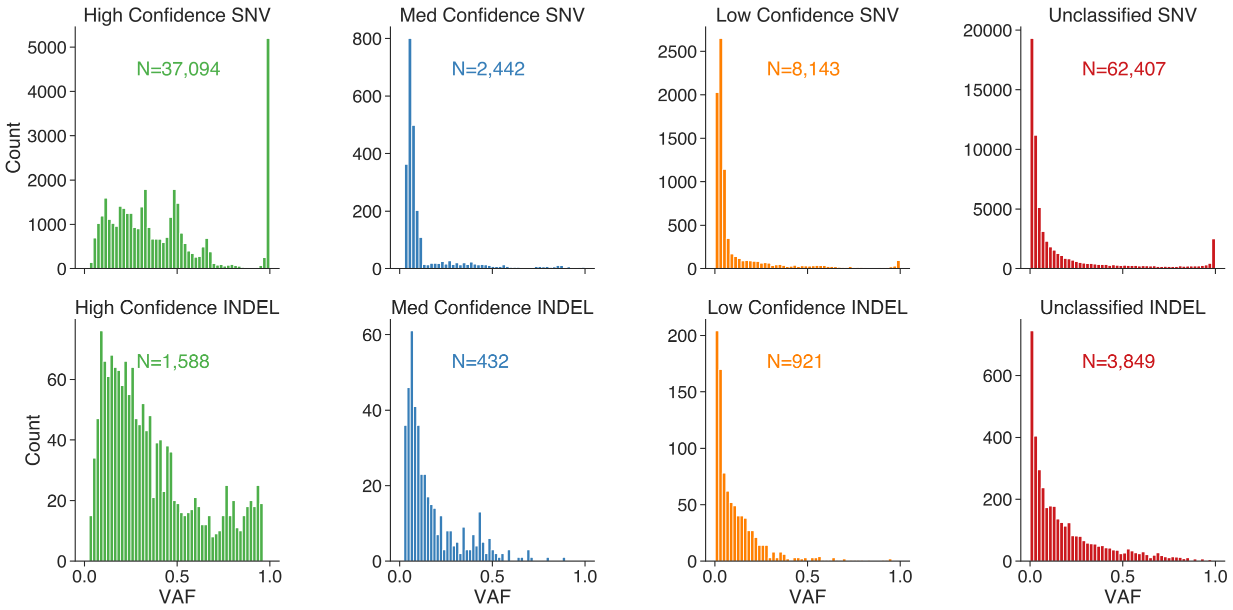

Fig. S1: VAF distribution of the ground truth SNV and INDEL somatic mutations in the super set of calls for HCC1395 classified by the SEQC2 consortium to four confidence levels (High, Med, Low, and Unclassified). High and medium confidence calls are grouped together as the “truth set” of somatic mutations.

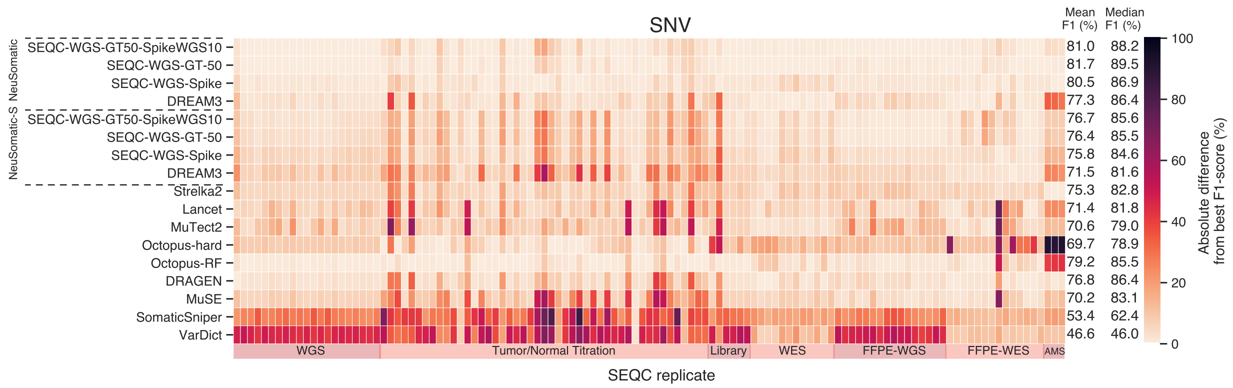

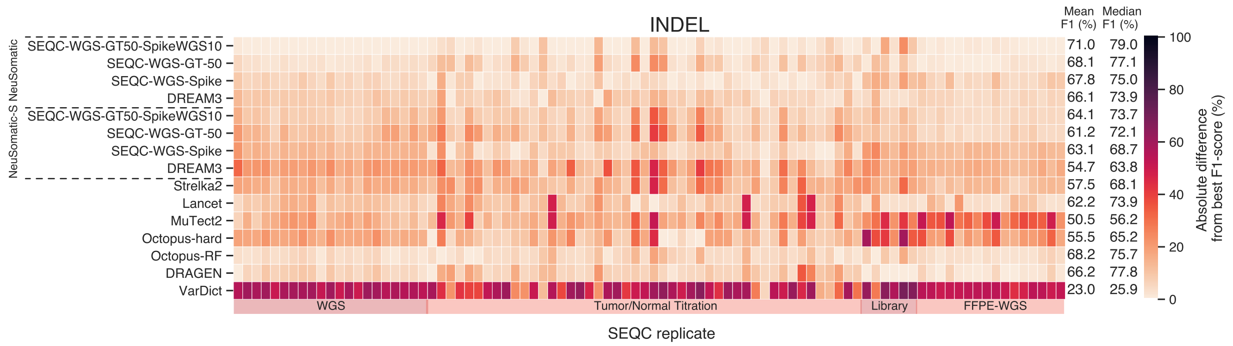

Fig. S2: Overall performance of different somatic mutation callers on 119 replicates in SEQC2 data set. The models trained on SEQC2 data achieved consistent superiority over other techniques across diverse sets of replicates of different purities/coverages in WGS, WES, FFPE, AmpliSeq (AMS), and different library prep data sets. In this figure, for each replicate the best F1-score was computed across different approaches. The heatmaps illustrate the absolute difference between the F1-score of any of the somatic mutation detection approaches to the best F1-score. In each panel, the mean F1-score is shown for each approach across 119 replicates.

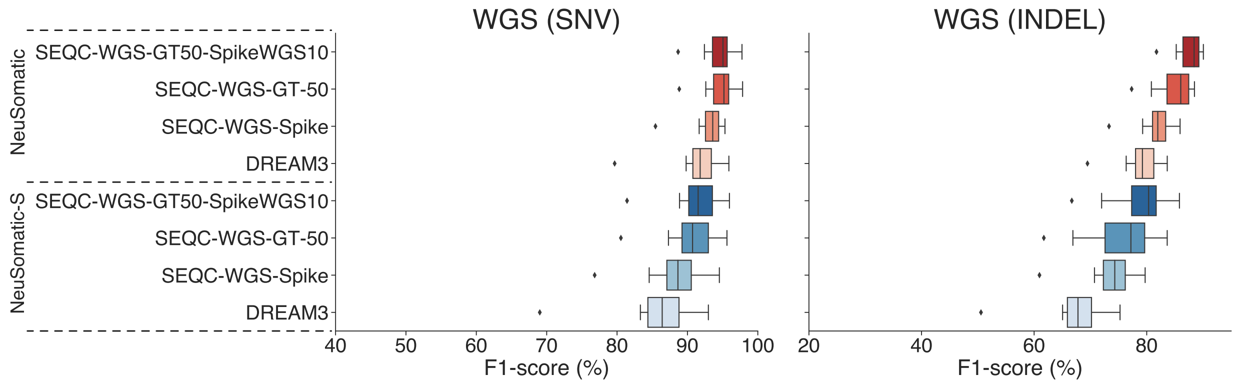

Fig. S3: Performance comparison of 8 different network models on WGS data sets: F1-score (%) comparison on 21 WGS replicates for SNVs and INDELs.

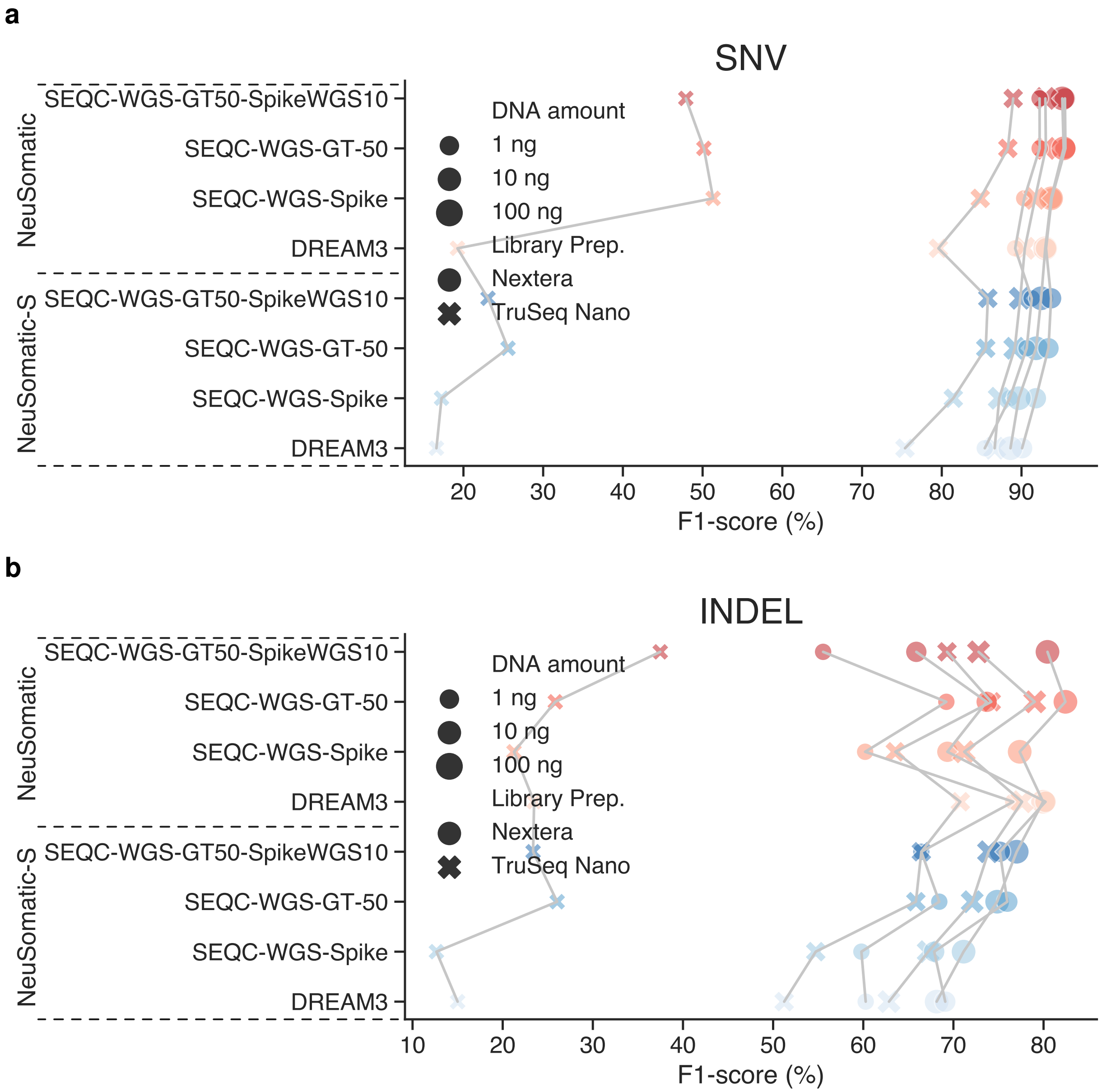

Fig. S4: Performance comparison of different network models on the library prep data sets: F1-score (%) comparison across different library kits and DNA amounts for (a) SNVs and (b) INDELs. Results of different models for the same library preparation kit and DNA amount are connected for better illustration.

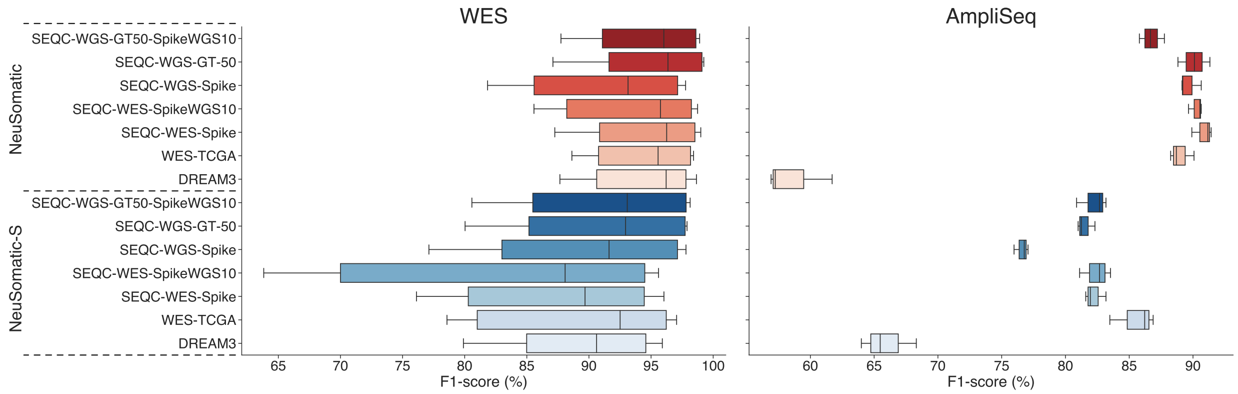

Fig. S5: Performance comparison of different network models on WES data sets: F1-score (%) comparison on 12 WES replicates for SNVs.

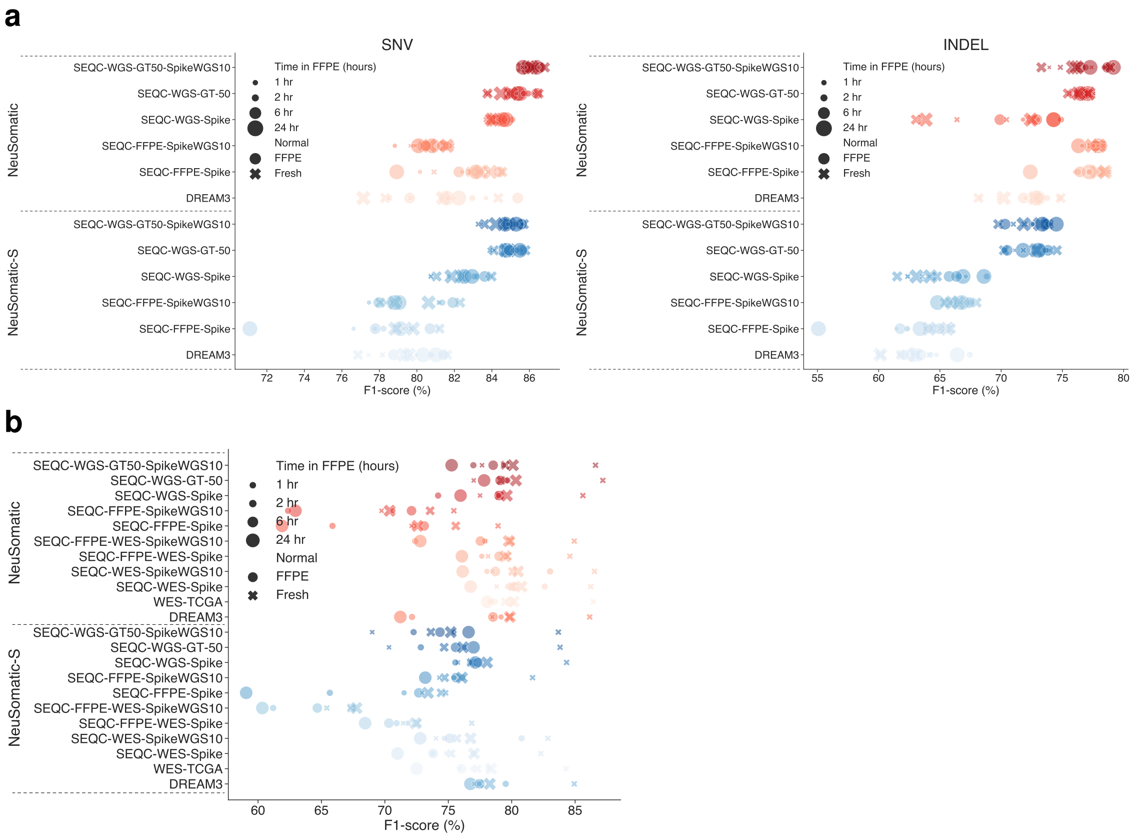

Fig. S6: Performance comparison of different network models on the FFPE data sets: F1-score (%) comparison across (a) 16 FFPE WGS replicates with FFPE and fresh matched normal, and (b) 14 FFPE WES replicates with FFPE and fresh matched normal.

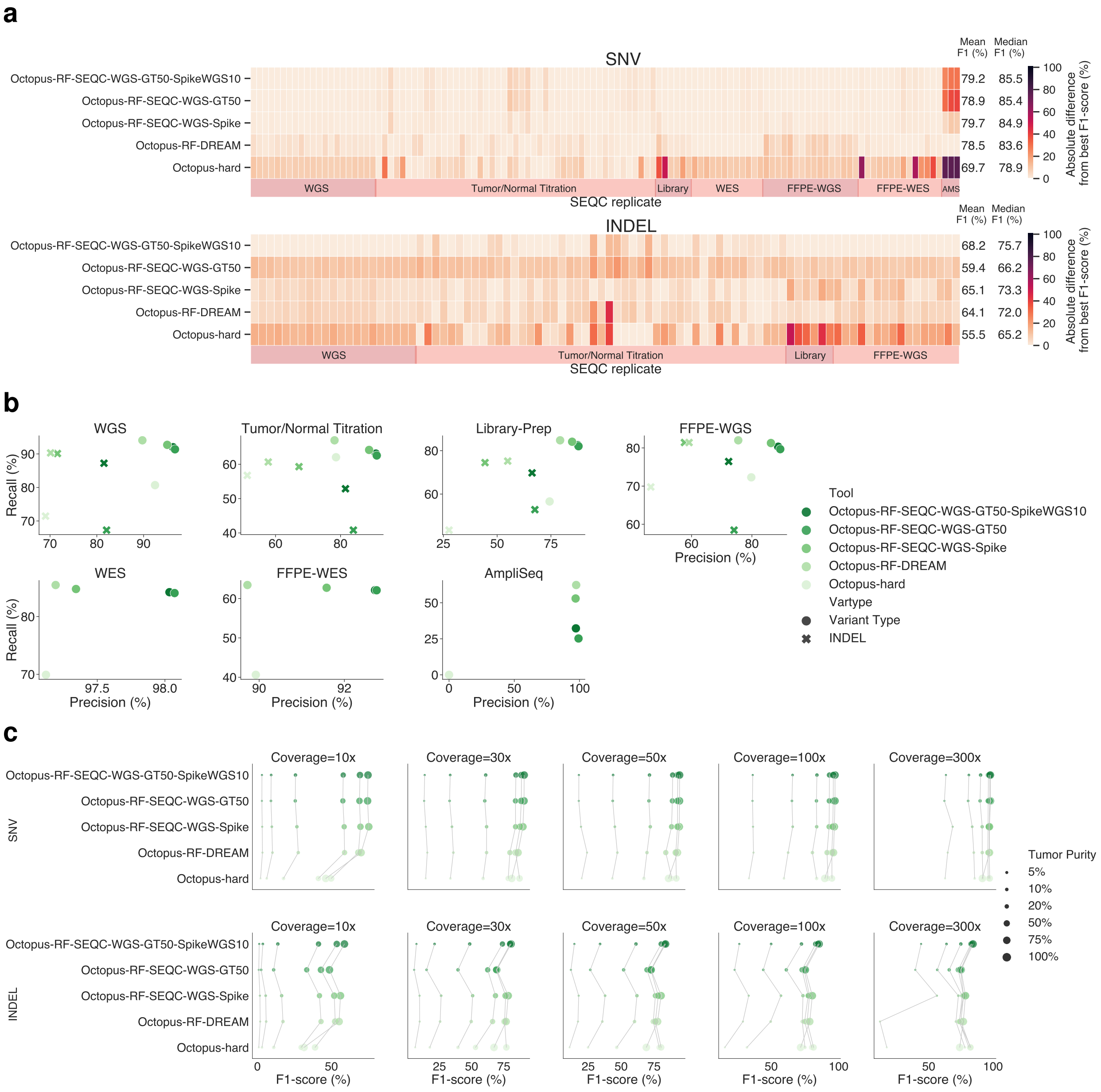

Fig. S7: Overall performance of different Octopus random forest filtering trained models as well as the hard-filtering (Octopus-hard) on 119 replicates in SEQC2 data set. (a) The models trained on SEQC2 data, achieved consistent superiority over the DREAM model across diverse sets of replicates of different purities/coverages in WGS, WES, FFPE, AmpliSeq (AMS), and different library preparation data sets. In this subfigure, for each replicate the best F1-score was computed across different approaches. The heatmaps illustrate the absolute difference between the F1-score of any of the models according to the best F1-score. In each panel, the mean F1-score is shown for each approach across 119 replicates. (b) Precision-Recall analysis of different models on different SEQC2 data sets. Each datapoint shows the average precision and recall values for SNVs and INDELs across the samples in a given dataset. (c) F1-score (%) comparison for different models across different coverages (10x-300x) and tumor purities (5%-100%). For a given coverage, results of different models on the same tumor purity are connected for better illustration.

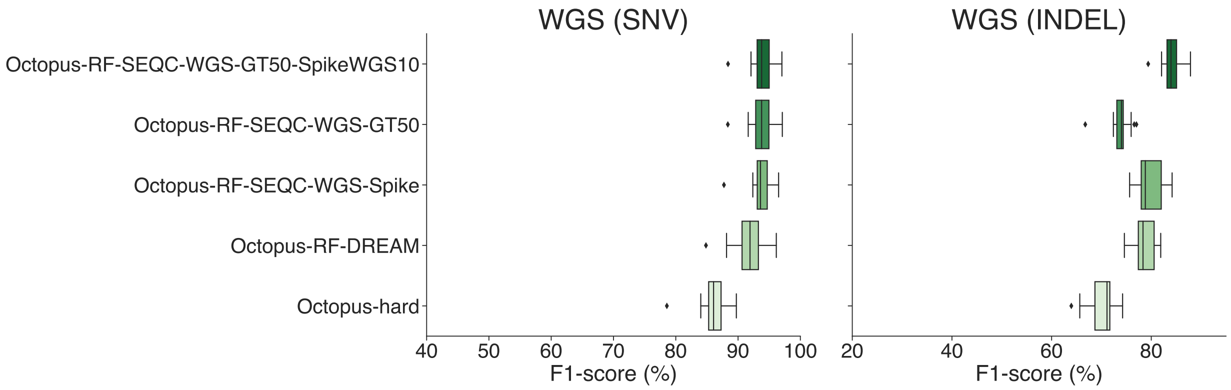

Fig. S8: Performance comparison of 8 different Octopus models on WGS data sets: F1-score (%) comparison on 21 WGS replicates for SNVs and INDELs.

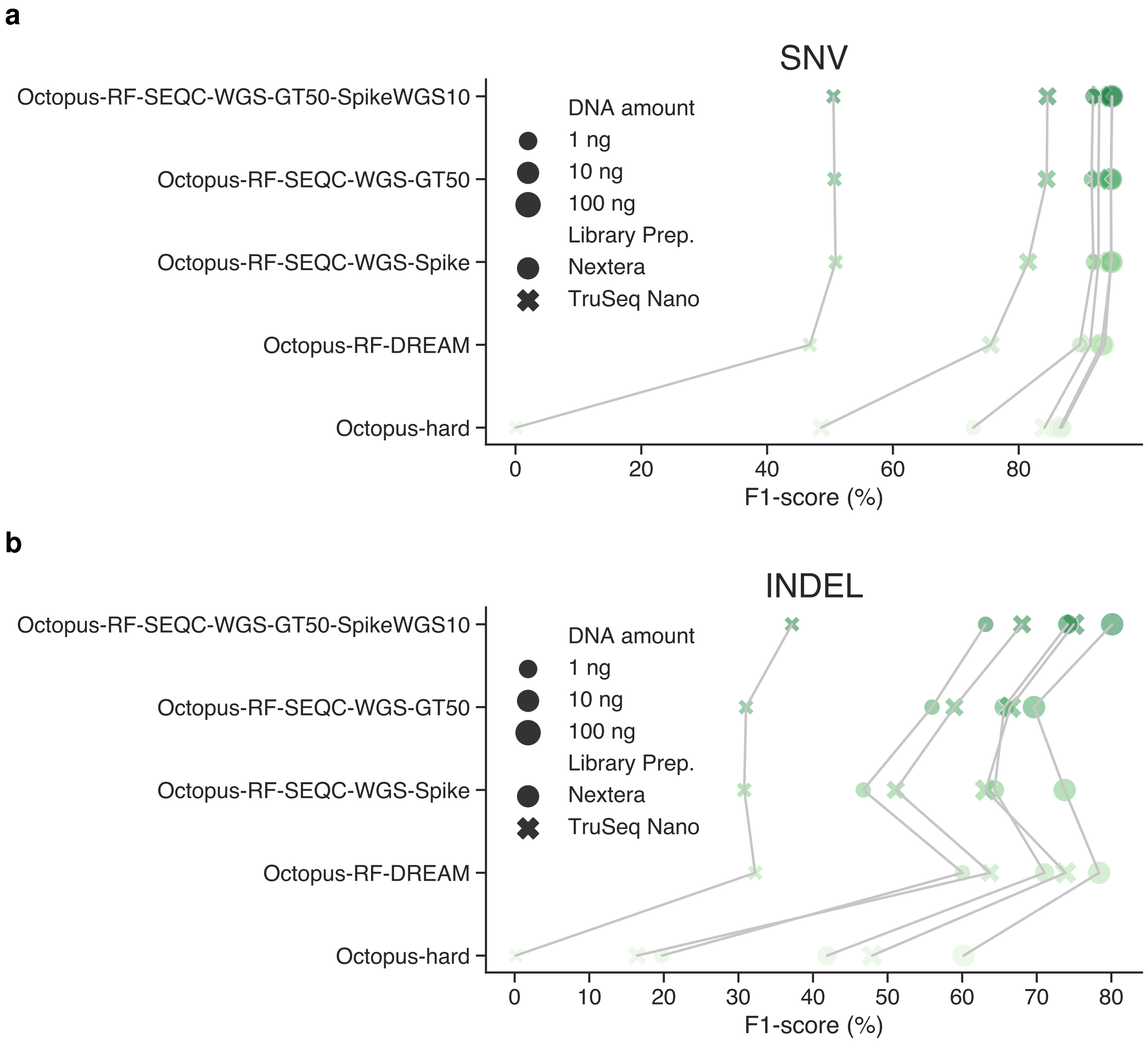

Fig. S9: Performance comparison of different Octopus models on the library prep data sets: F1-score (%) comparison across different library kits and DNA amounts for (a) SNVs and (b) INDELs. Results of different models for the same library preparation kit and DNA amount are connected for better illustration.

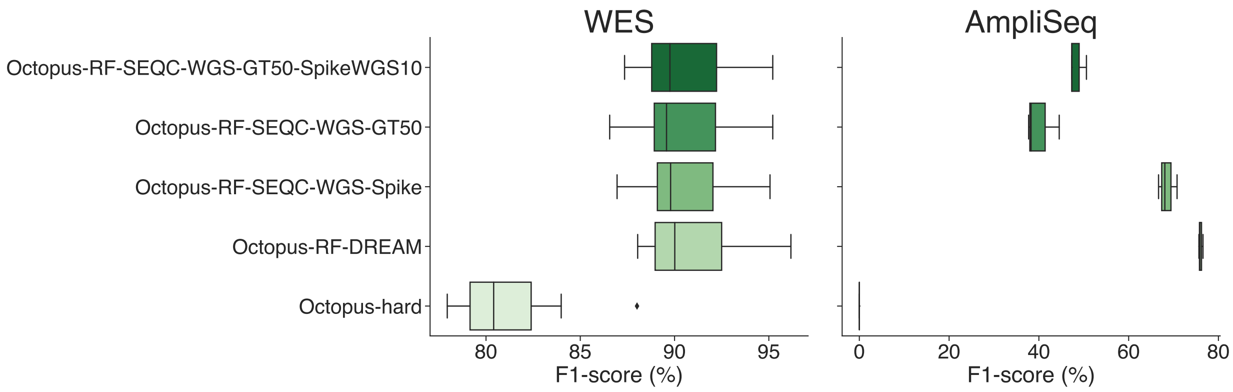

Fig. S10: Performance comparison of different Octopus models on WES data sets: F1-score (%) comparison on 12 WES replicates for SNVs.

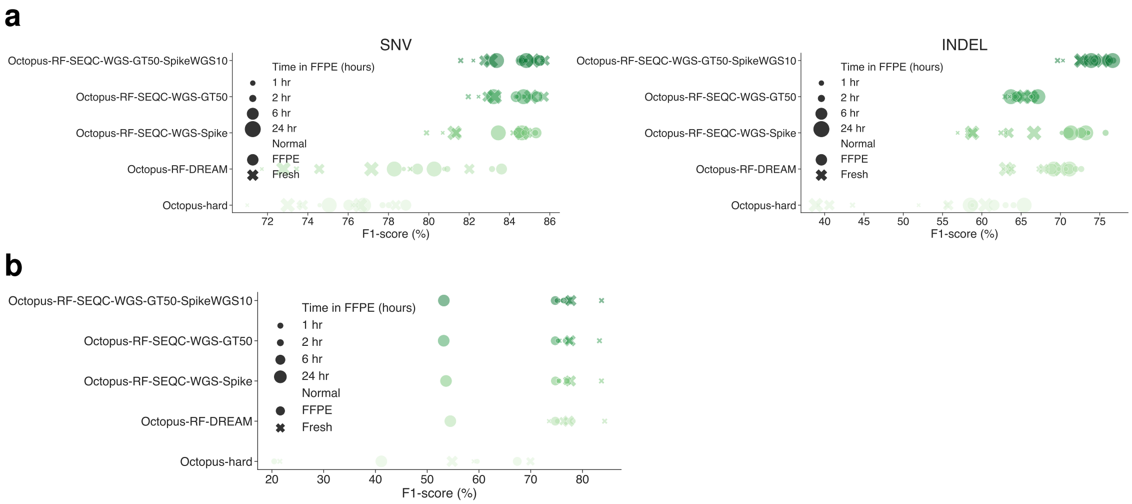

Fig. S11: Performance comparison of different Octopus models on the FFPE data sets: F1-score (%) comparison across (a) 16 FFPE WGS replicates with FFPE and fresh matched normal, and (b) 14 FFPE WES replicates with FFPE and fresh matched normal.

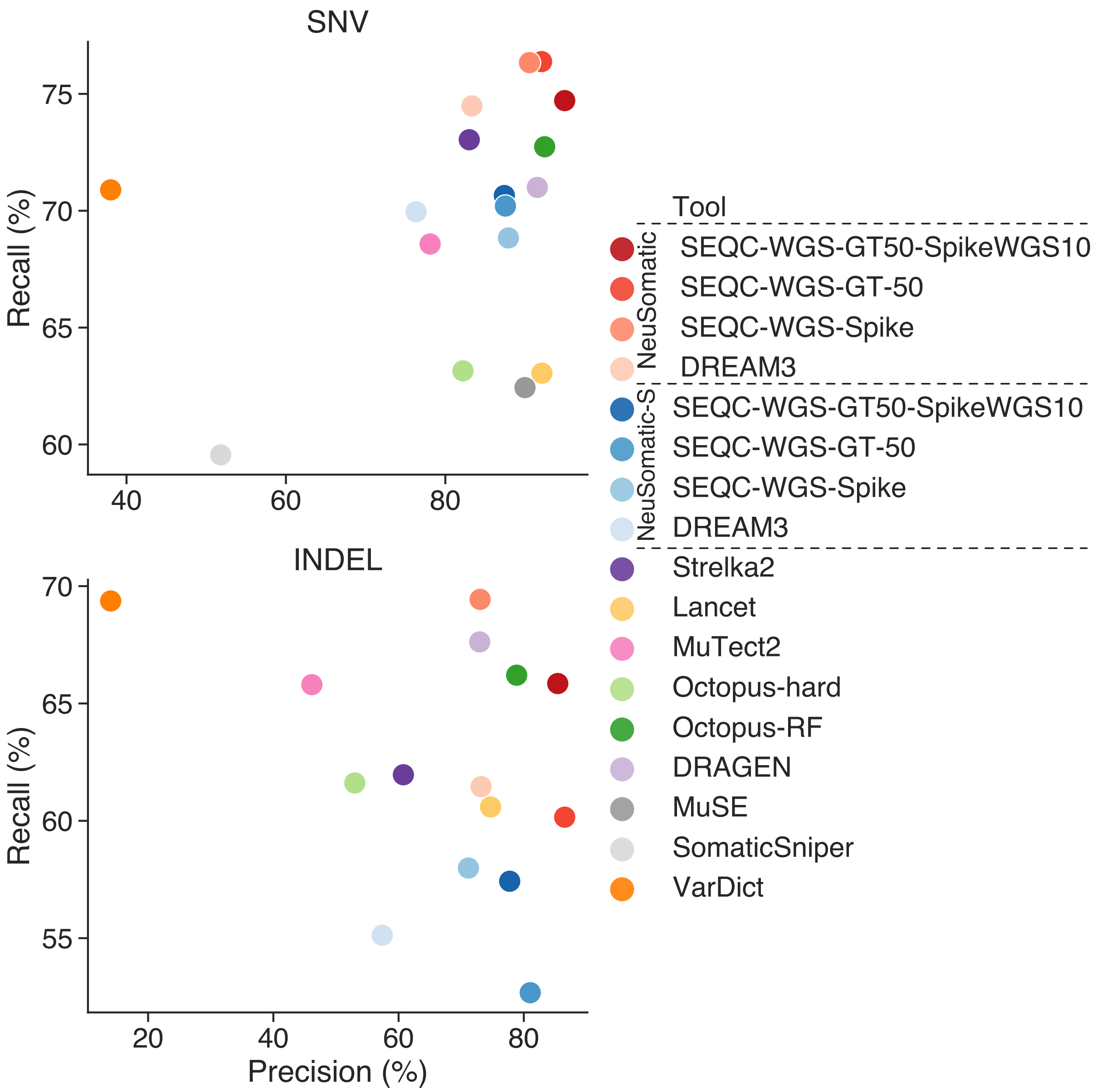

Fig. S12: Precision-Recall analysis of different somatic mutation callers on 119 replicates in SEQC2 data set. Each datapoint shows the average precision and recall values across all replicates.

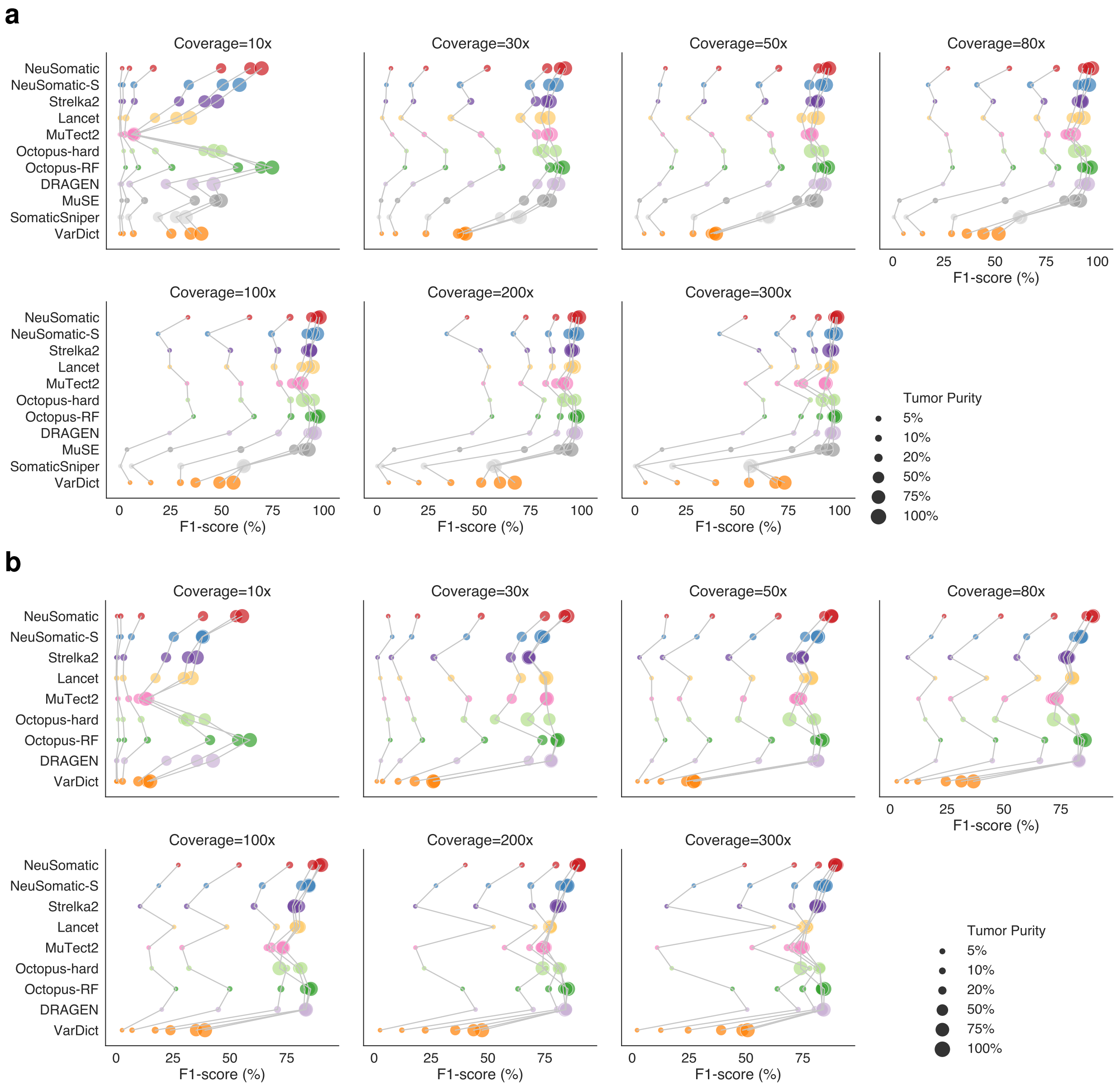

Fig. S13: F1-score (%) comparison for different somatic mutation callers across different coverages (10x-300x) and tumor purities (5%-100%): (a) SNVs, (b) INDELs. For a given coverage, results of different callers on the same tumor purity are connected for better illustration.

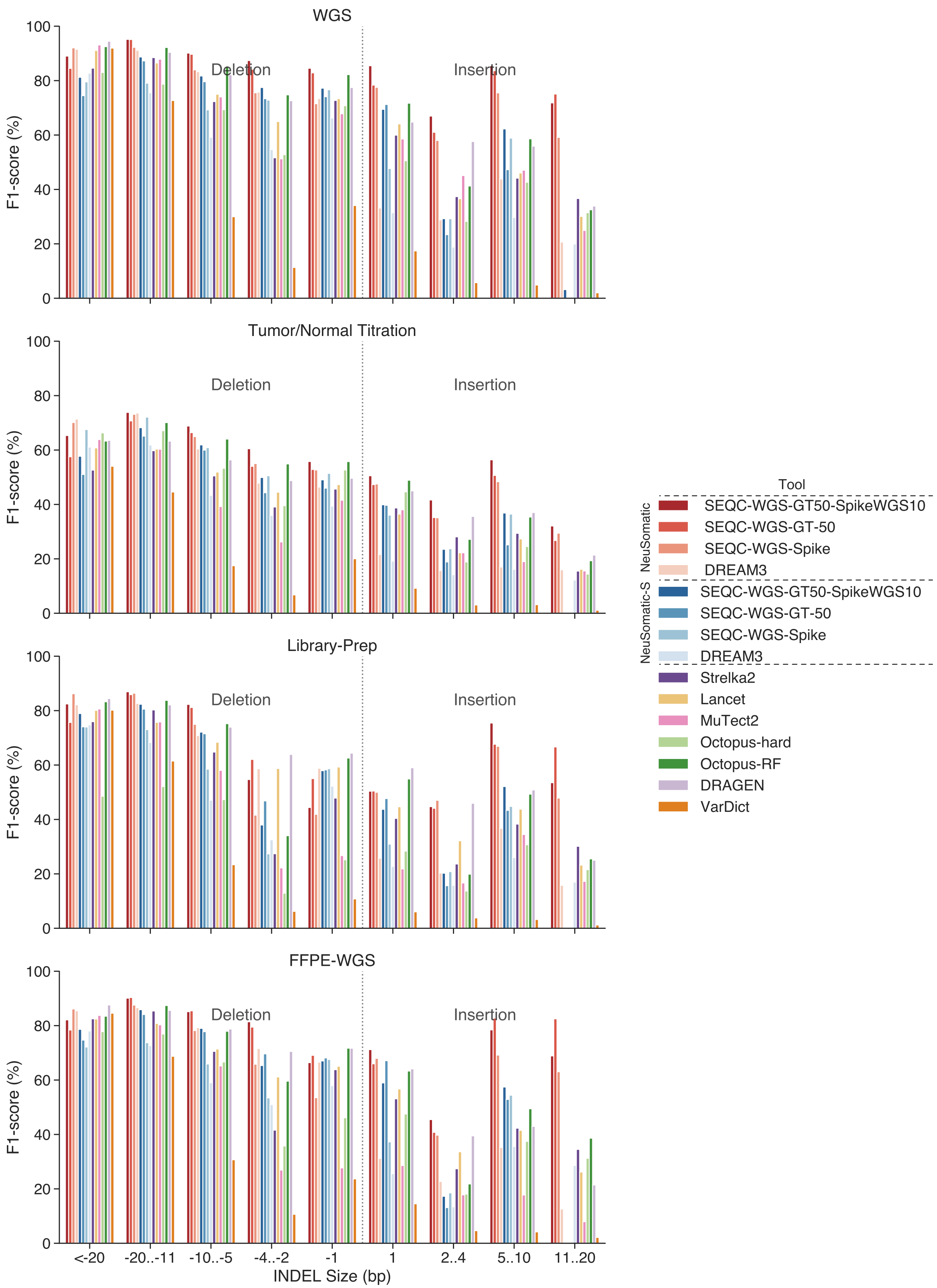

Fig. S14: Average performance of somatic mutation callers for different INDEL sizes on SEQC2 data sets. Negative INDEL sizes reflect deletion.

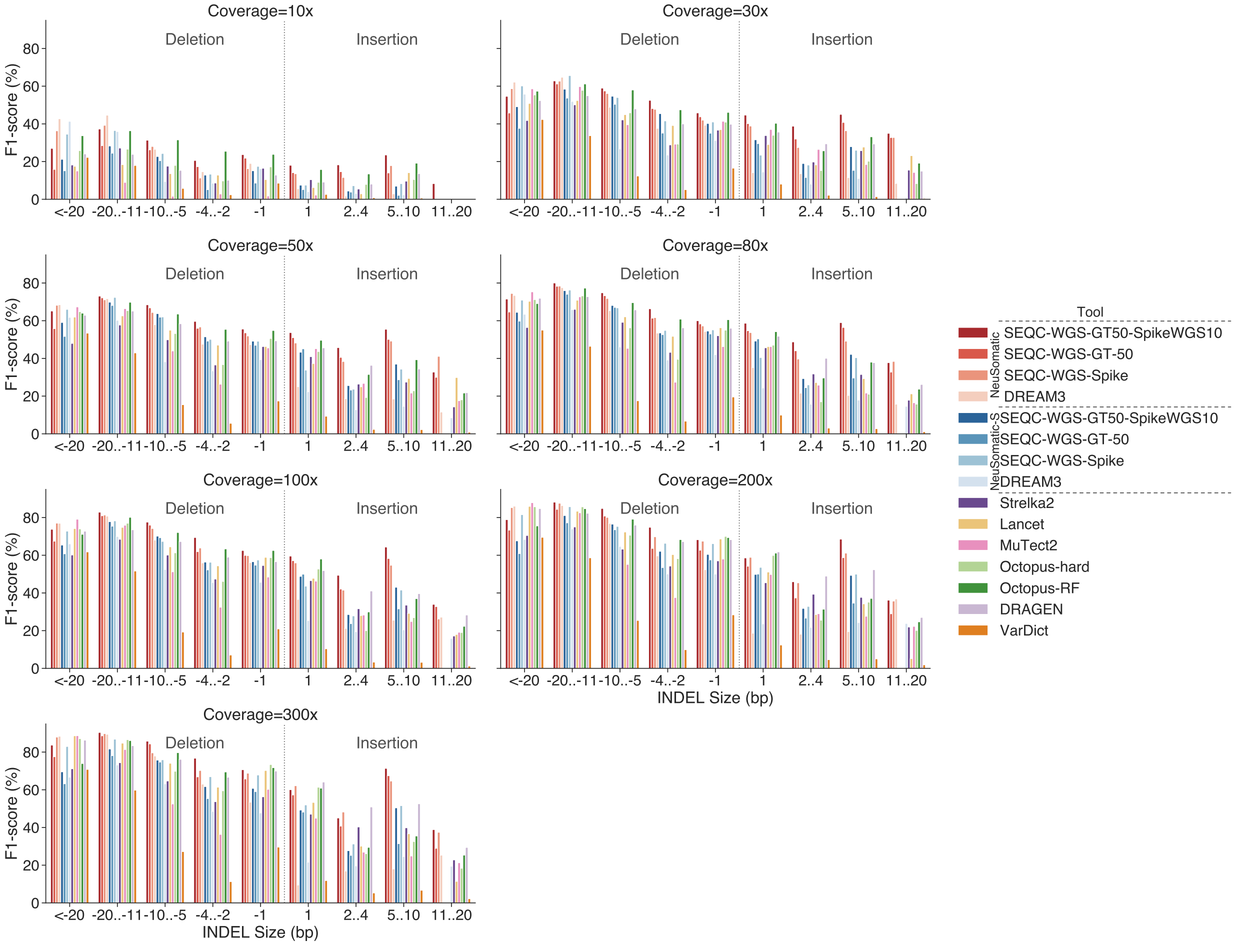

Fig. S15: Average performance of somatic mutation callers for different INDEL sizes and coverages ranging from 10x to 300x in the Tumor/Normal titration data set. Negative INDEL sizes reflect deletions.

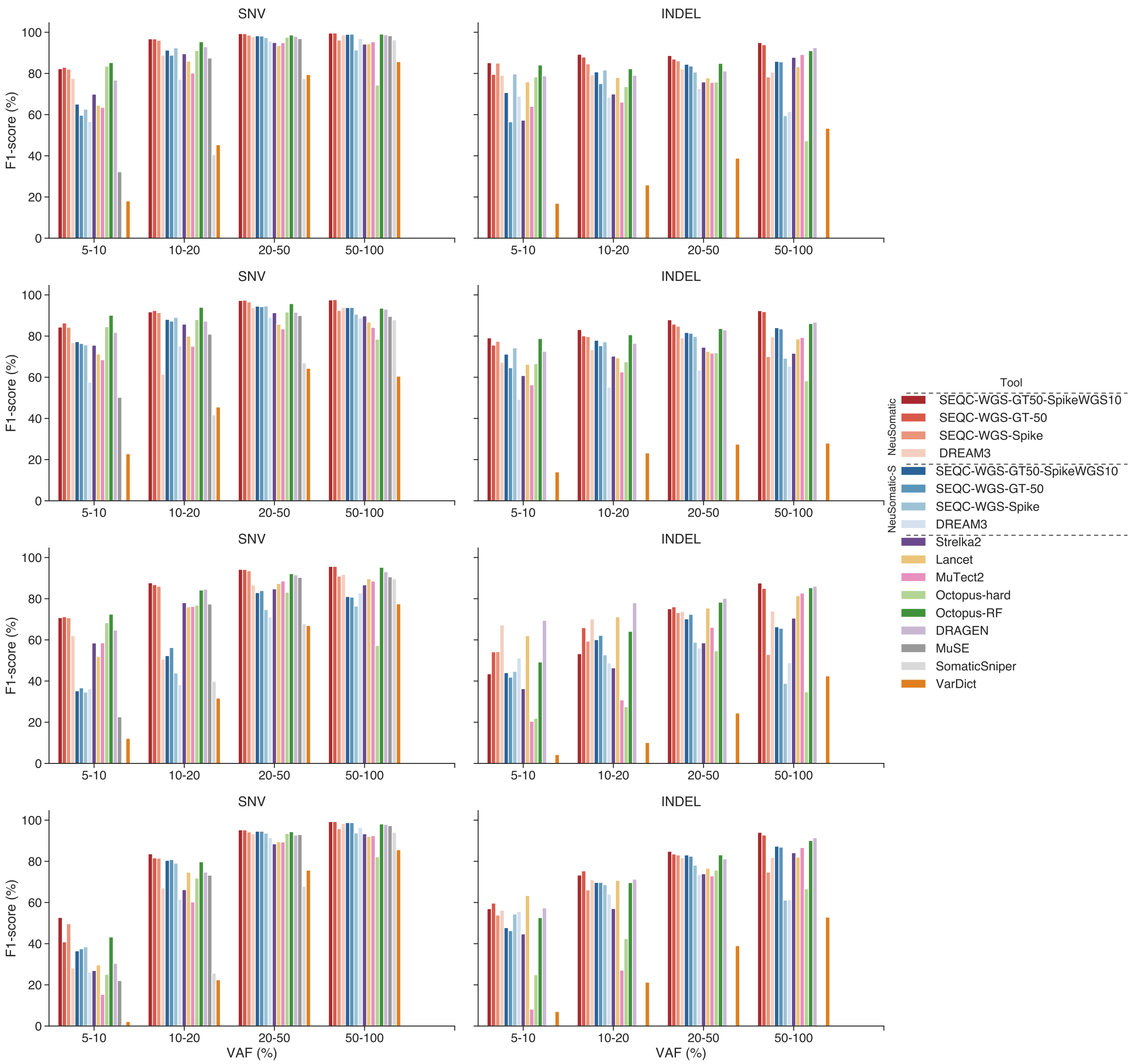

Fig. S16: Performance analysis for mutations with different VAF ranges across SEQC2 data sets using different callers.

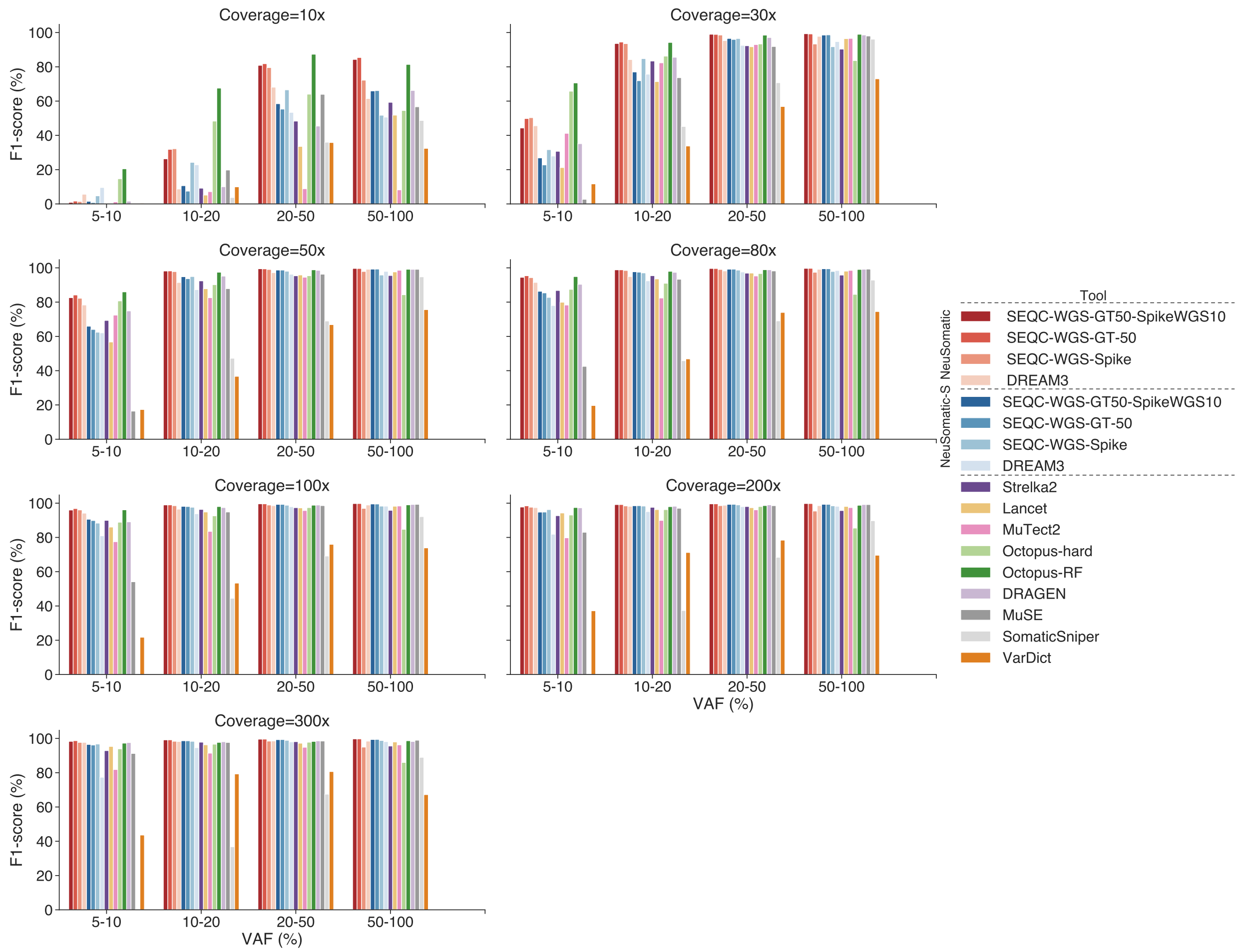

Fig. S17: Performance analysis for SNVs with different VAF ranges on Tumor/Normal titration data set using different callers.

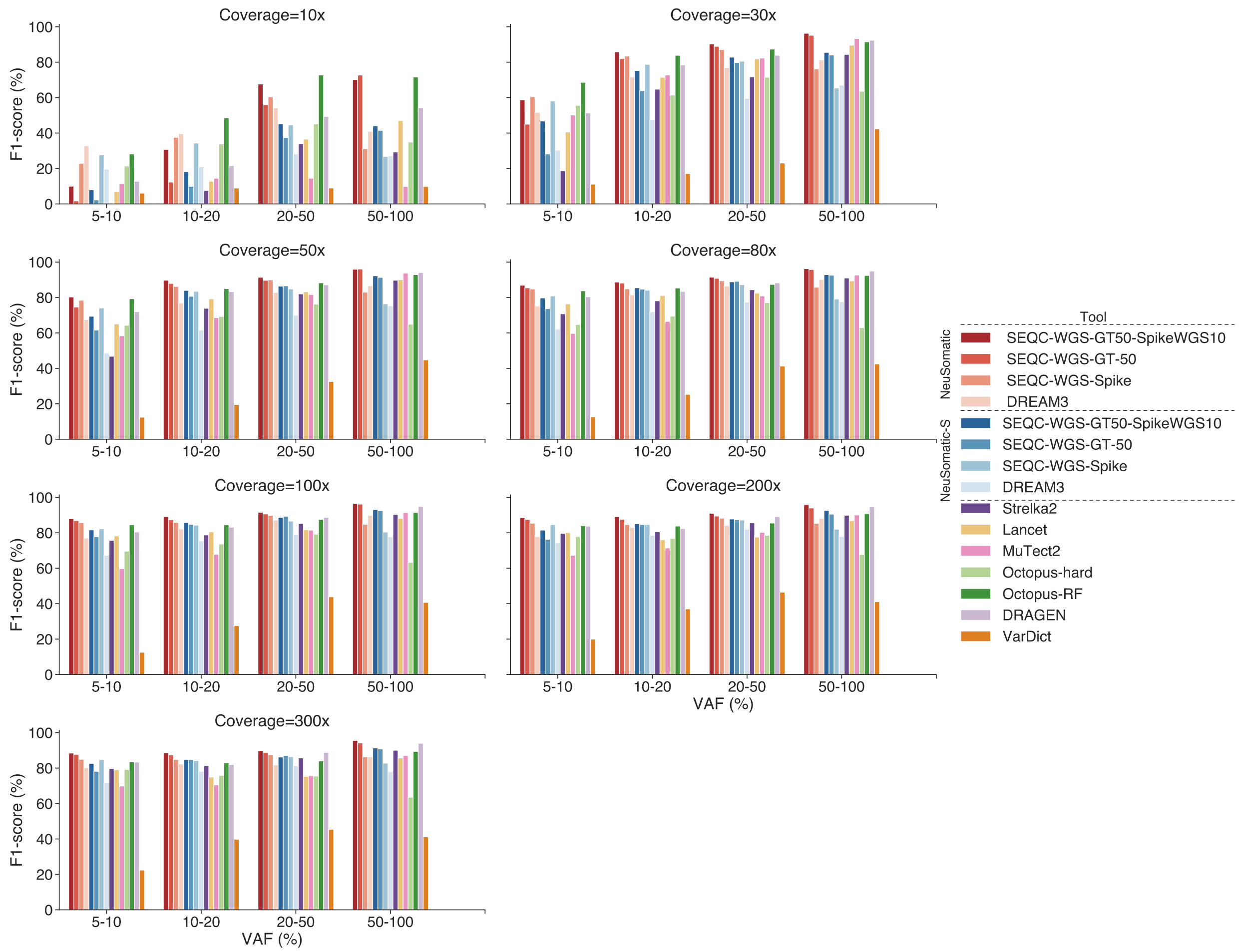

Fig. S18: Performance analysis for INDELs with different VAF ranges on Tumor/Normal titration data set using different callers.

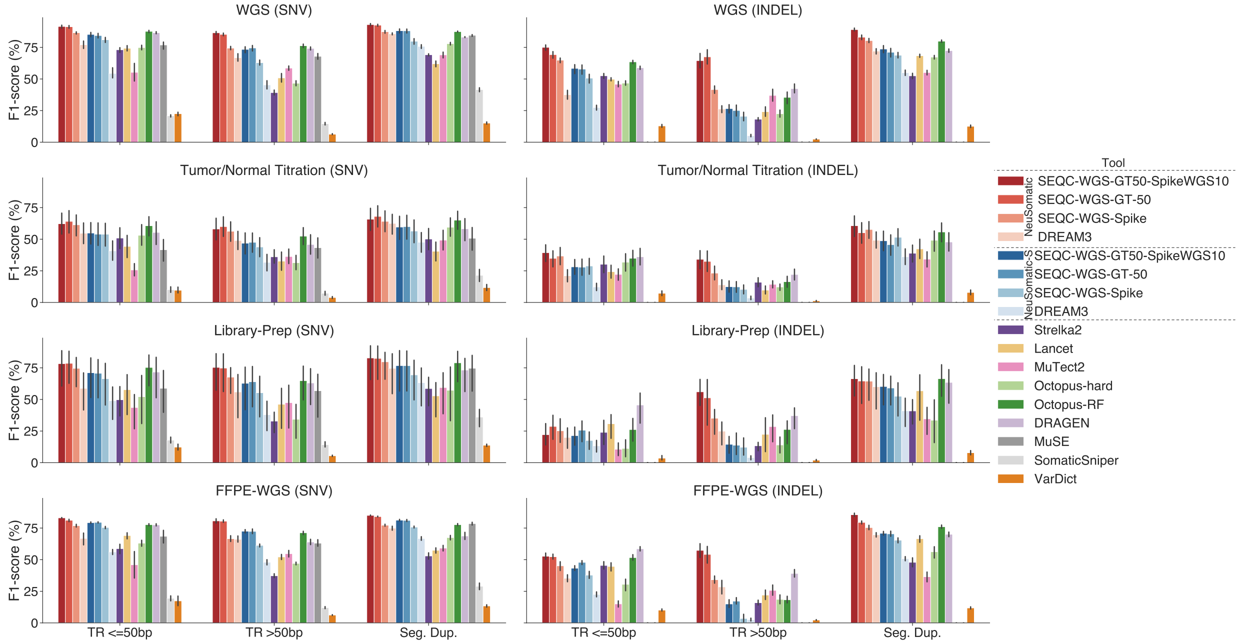

Fig. S19: Performance analysis of somatic mutation callers on difficult regions including tandem repeats (TR) of different sizes and segmental duplications for different SEQC2 data sets.

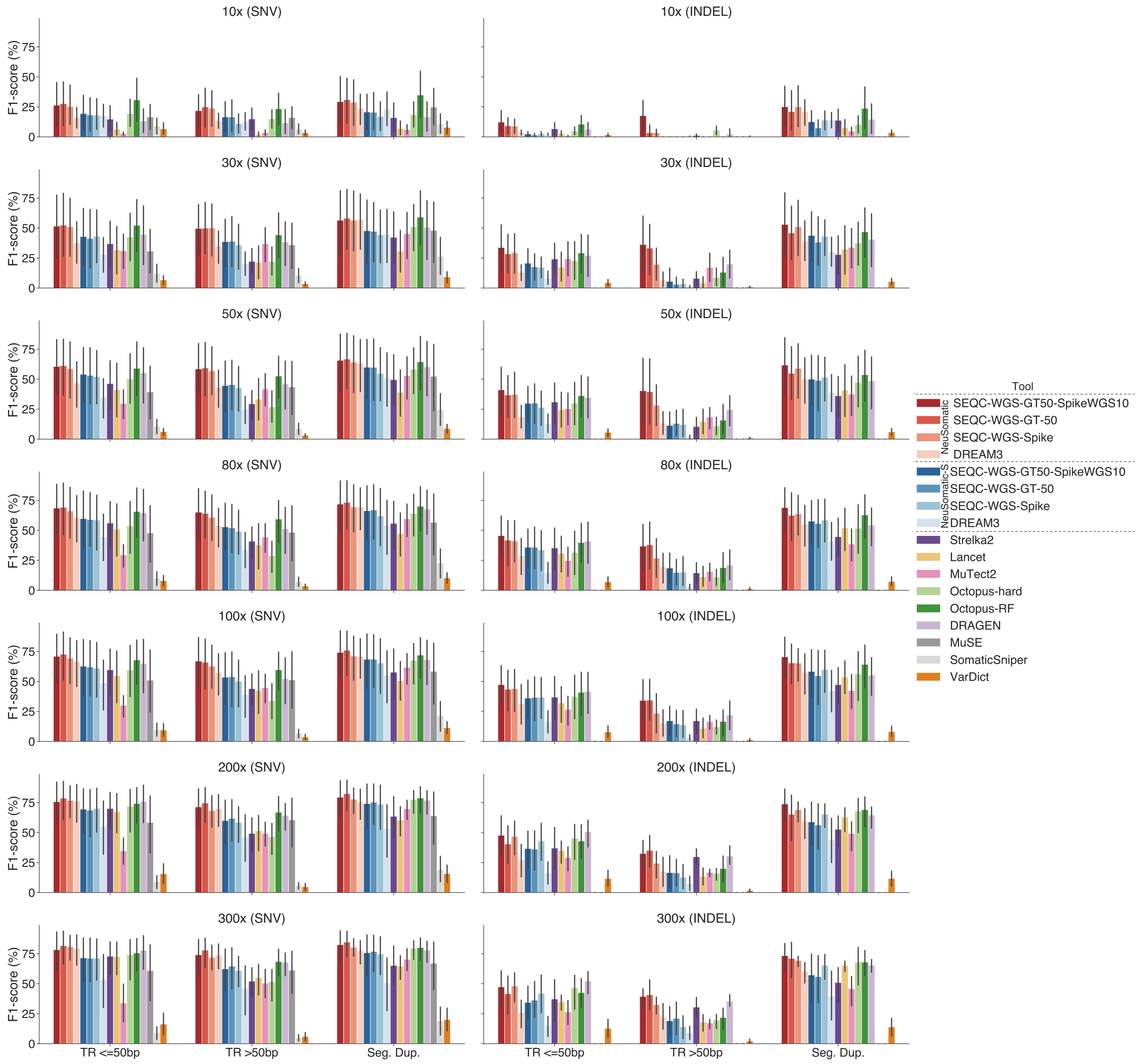

Fig. S20: Performance analysis of somatic mutation callers on difficult regions including tandem repeats (TR) of different sizes and segmental duplications for Tumor/Normal titration data set using different callers.

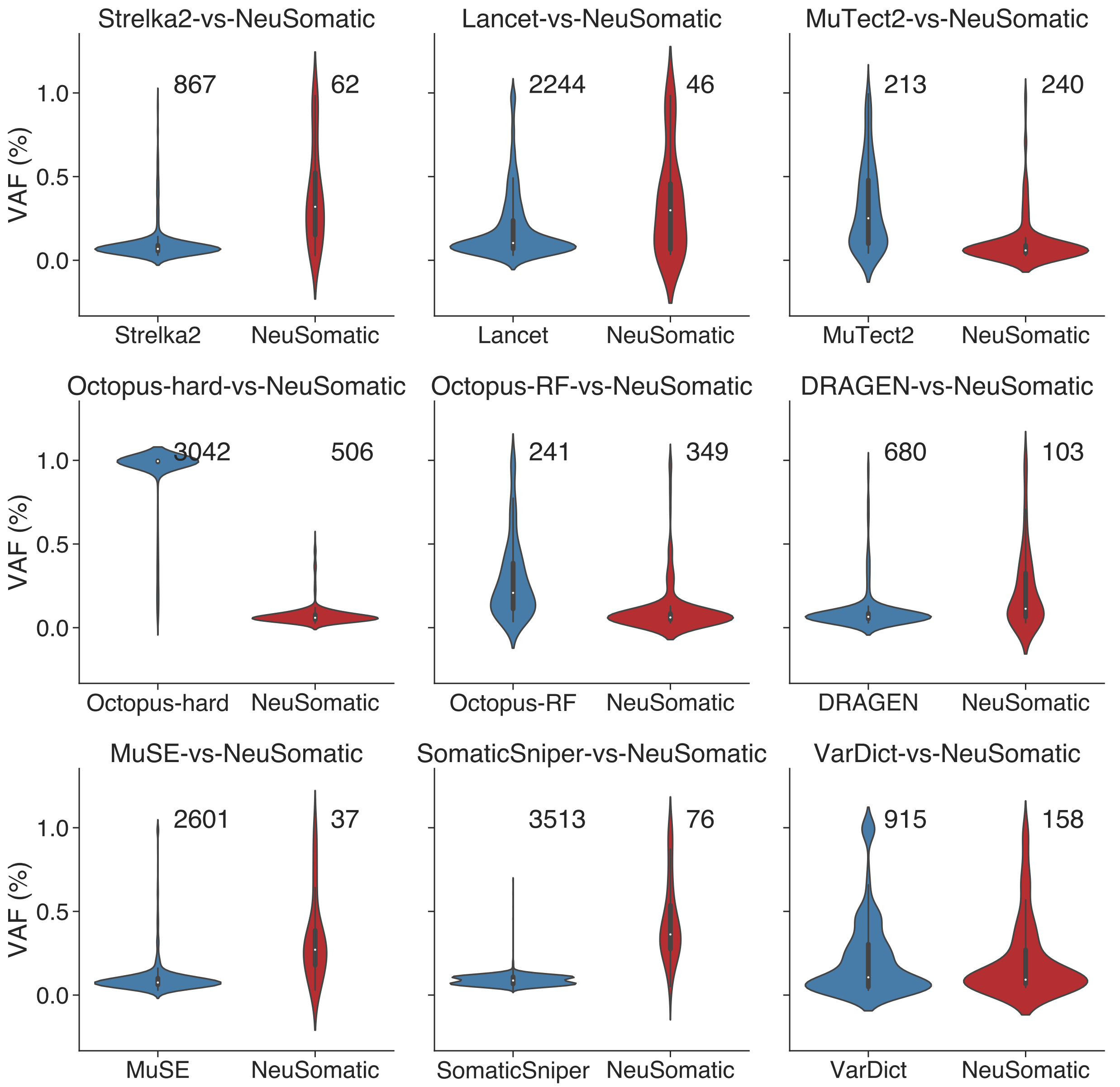

Fig. S21: The violin plot comparison of the VAF distribution of private false negative (FN) calls on WGS data set. In each subfigure we compared one of the conventional somatic mutation detection schemes against NeuSomatic. For an X-vs-Y subfigure, we identified X_{FN}, the set of ground truth SNVs which were missed by algorithm X (FN in X) in at least 11 out of 21 WGS replicates. Similarly, we identified Y_{FN}, the set of ground truth SNVs which were missed by algorithm Y (FN in Y) in at least 11 out of 21 WGS replicates. The figure then shows the VAF distribution of private FN calls for X and Y. In other words, the violin plot shows the VAF distribution of calls in the set $X_{\text{FN}}/Y_{\text{FN}}$ in blue and the VAF distribution of calls in the set $Y_{\text{FN}}/X_{\text{FN}}$ in red. For most of the conventional schemes like Strelka2, the private FNs, which were correctly predicted by NeuSomatic, had low VAF which revealed the inferiority of such approaches in detecting low VAF mutations. In each X-vs-Y subfigure, the number of private FNs for X and Y are reported on the top.

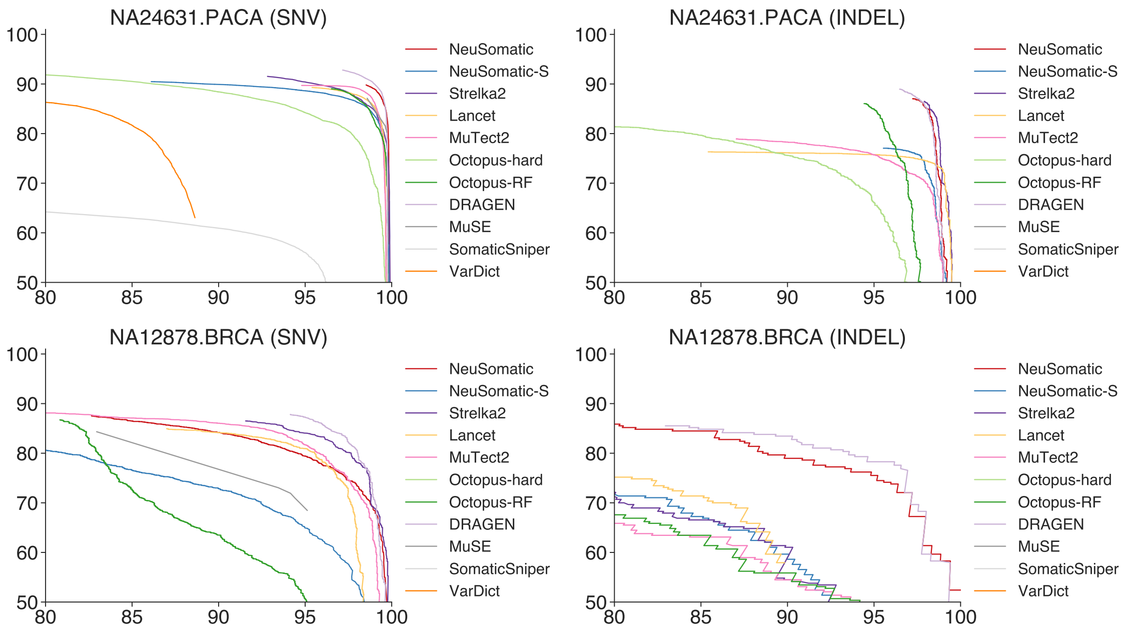

Fig. S22: Precision-Recall analysis of different somatic mutation callers on synthetic tumor samples NA24631.PACA and NA12878.BRCA.
