# Supplementary material for: Achieving robust somatic mutation detection with deep learning models derived from reference data sets of a cancer sample
Source: Genome Biol. 2022 Jan 7;23:12. doi: 10.1186/s13059-021-02592-9 (PMC8740374; doi:10.1186/s13059-021-02592-9)
Supplement: Supplementary file 2 — Additional file 2:. Supplementary Tables. [file 13059_2021_2592_MOESM2_ESM.pdf]

Sahraeian et al. Achieving robust somatic mutation detection with deep learning models derived from reference data sets of a cancer sample

Supplementary Tables

Table S1 -Trained Network models used in this study

**Model**	**Training Dataset**	**Somatic mutations**	**Region Trained**	**# of T/N replicates**	**Coverage of samples**	**Somatic mutations AF**	**# of somatic mutations in all replicates**	**# of total candidate variants used for training**
DREAM3	DREAM3 WGS	*In silico* spikes	50% of genome	5	30x	5-50%	450K	29.2M
WES-TCGA	TCGA	*In silico* spikes	exome	12	45x-185x	0.5-100%	1.5M	5.9M
SEQC-WGS-Spike	SEQC2 WGS	*In silico* spikes	genome	20	40x-170x	0.3-90%	2M	272M
SEQC-WGS-GT-50	SEQC2 WGS	Real GT mutations in SEQC2	50% of genome	24	40x-390x	0.5-100%	416K	137M
SEQC-WGS-GT50-SpikeWGS10	SEQC2 WGS	Real GT mutations in SEQC2 + in silico spikes from 10% of WGS	genome	44	40x-390x	0.3-100%	574K	164M
SEQC-WES-Spike	SEQC2 WES	*In silico* spikes	exome	7	60x-550x	0.1-100%	755K	3.7M
SEQC-FFPE-Spike	SEQC2 WGS-FFPE	*In silico* spikes	genome	16	54x-77x	0.5-83%	1.7M	191M
SEQC-FFPE-WES-Spike	SEQC2 WES-FFPE	*In silico* spikes	exome	14	70x-90x	0.1-88%	1.4M	9.6M
SEQC-WES-SpikeWGS10	SEQC2 WES/WGS	*In silico* spikes	exome	27	40x-550x	0.1-100%	963K	30.9M
SEQC-FFPE-SpikeWGS10	SEQC2 WGS-FFPE/WGS	*In silico* spikes	genome	36	40x-170x	0.3-90%	1.9M	218M
SEQC-FFPE-WES-SpikeWGS10	SEQC2 WES-FFPE/WGS	*In silico* spikes	exome	47	40x-170x	0.1-90%	1.6M	36.8M

Table S2 - F1-score (%) performance of different somatic mutation detection methods and network trained models on the WGS data set

Tumor	Normal	Tool																
											NeuSomatic-S				NeuSomatic			
		VarDict	SomaticSniper	MuSE	DRAGEN	Octopus-RF	Octopus-hard	MuTect2	Lancet	Strelka2	DREAM3	SEQC-WGS-Spike	SEQC-WGS-GT-50	SEQC-WGS-GT50-SpikeWGS10	DREAM3	SEQC-WGS-Spike	SEQC-WGS-GT-50	SEQC-WGS-GT50-SpikeWGS10
**SNVs**																		
WGS_EA_T_1	WGS_EA_N_1	48.1	65.1	90.2	93.8	95.4	87.3	90.8	90.3	91.1	89.4	91.7	93.4	93.7	93.8	94.5	96.1	96.0
WGS_FD_T_1	WGS_FD_N_1	46.8	68.4	88.0	91.3	93.7	85.8	90.5	87.9	88.6	86.8	87.7	89.5	90.2	91.8	92.6	93.7	93.6
WGS_FD_T_2	WGS_FD_N_2	46.6	67.5	88.6	91.8	93.8	85.8	90.7	88.6	88.8	86.8	88.5	90.1	90.8	91.8	93.0	94.0	94.1
WGS_FD_T_3	WGS_FD_N_3	46.7	66.8	88.4	91.7	93.7	85.5	90.5	88.6	88.7	86.4	88.5	90.2	90.9	91.8	92.8	93.9	93.9
WGS_IL_T_1	WGS_IL_N_1	53.5	67.5	90.6	94.6	96.2	88.3	91.9	91.1	91.3	90.0	91.2	93.3	93.9	95.0	95.0	96.8	96.6
WGS_IL_T_2	WGS_IL_N_2	47.8	68.5	89.0	92.9	94.9	86.3	90.5	89.0	89.4	88.8	89.3	91.4	92.0	93.3	93.6	95.3	95.2
WGS_IL_T_3	WGS_IL_N_3	49.1	68.6	88.7	92.9	94.8	86.6	90.3	88.4	89.4	88.7	88.7	90.7	91.5	93.4	93.7	95.2	95.1
WGS_LL_T_1	WGS_LL_N_1	43.6	69.7	80.6	91.3	88.4	78.6	79.0	86.4	79.7	69.0	76.8	80.5	81.4	79.7	85.5	88.8	88.7
WGS_NC_T_1	WGS_NC_N_1	42.3	68.2	88.0	91.4	93.5	85.3	89.5	87.1	88.3	87.4	87.1	89.1	89.9	91.5	92.6	93.7	93.6
WGS_NS_T_1	WGS_NS_N_1	49.8	67.4	88.4	92.7	94.6	86.9	79.2	87.0	89.6	84.8	89.9	92.6	93.2	91.7	94.2	95.7	95.6
WGS_NS_T_2	WGS_NS_N_2	48.4	69.1	85.9	89.8	92.1	84.4	84.1	80.5	87.3	84.4	85.1	87.6	89.0	90.6	91.7	92.7	92.4
WGS_NS_T_3	WGS_NS_N_3	44.6	68.1	87.6	91.2	93.1	84.0	83.8	85.5	87.9	84.3	87.1	89.8	90.9	90.8	92.6	93.9	93.8
WGS_NS_T_4	WGS_NS_N_4	49.4	68.2	87.6	92.3	94.1	86.7	79.0	85.8	89.4	83.4	89.6	92.3	92.9	90.8	93.9	95.6	95.5
WGS_NS_T_5	WGS_NS_N_5	47.4	67.3	84.8	89.6	92.7	84.8	78.9	78.9	87.8	84.5	86.9	89.2	90.2	90.5	92.3	93.4	93.2
WGS_NS_T_6	WGS_NS_N_6	50.6	67.3	88.4	92.8	95.0	88.1	79.0	85.7	89.9	86.1	90.5	93.0	93.5	92.4	94.4	95.9	95.6
WGS_NS_T_7	WGS_NS_N_7	50.3	68.3	84.9	90.7	93.6	86.1	73.4	80.9	88.8	83.3	89.3	91.7	92.3	89.8	93.6	95.3	95.1
WGS_NS_T_8	WGS_NS_N_8	49.0	69.3	86.2	89.8	92.1	84.5	85.8	81.8	87.2	84.4	84.6	87.3	88.9	90.7	91.7	92.6	92.4
WGS_NS_T_9	WGS_NS_N_9	47.6	68.8	87.2	90.8	92.7	85.7	86.5	83.6	87.8	84.8	86.0	88.6	89.9	91.2	92.3	93.5	93.3
WGS_NV_T_1	WGS_NV_N_1	55.5	62.7	92.2	95.9	97.0	89.3	88.2	93.8	92.9	92.9	94.5	95.5	96.0	95.7	95.3	97.8	97.8
WGS_NV_T_2	WGS_NV_N_2	55.6	62.7	92.3	95.9	97.0	89.3	87.8	93.9	93.1	93.0	94.4	95.6	95.9	95.7	95.3	97.8	97.7
WGS_NV_T_3	WGS_NV_N_3	57.3	63.4	92.2	95.7	97.0	89.7	92.0	93.8	93.0	92.8	94.5	95.6	95.9	95.9	95.3	97.8	97.7
Average		49.0	67.3	88.1	92.3	94.1	86.1	85.8	87.1	89.0	86.3	88.7	90.8	91.6	91.8	93.1	94.7	94.6
**INDELs**																		
WGS_EA_T_1	WGS_EA_N_1	31.7	-	-	80.5	83.2	68.0	71.0	76.2	75.1	71.7	76.2	80.3	81.7	81.2	81.2	88.0	89.9
WGS_FD_T_1	WGS_FD_N_1	28.6	-	-	78.8	83.0	66.6	73.7	77.3	72.1	66.0	74.3	76.5	79.7	78.5	80.6	86.7	88.4
WGS_FD_T_2	WGS_FD_N_2	29.6	-	-	78.5	82.5	65.7	72.7	77.6	72.4	66.4	74.4	77.2	80.3	77.8	81.7	87.7	88.7
WGS_FD_T_3	WGS_FD_N_3	28.7	-	-	78.9	82.1	65.8	74.1	77.5	71.8	65.1	74.2	77.6	80.9	78.8	79.3	87.5	88.5
WGS_IL_T_1	WGS_IL_N_1	37.5	-	-	86.3	87.9	74.2	80.6	83.4	77.3	71.1	77.6	78.0	81.6	81.4	84.8	86.0	89.0
WGS_IL_T_2	WGS_IL_N_2	33.2	-	-	83.2	85.5	71.7	80.1	81.7	73.6	68.4	74.1	76.4	79.5	79.7	82.1	85.0	88.3
WGS_IL_T_3	WGS_IL_N_3	32.6	-	-	83.9	85.1	71.4	80.3	80.7	73.7	67.8	72.3	73.1	78.1	79.2	82.7	83.6	86.4
WGS_LL_T_1	WGS_LL_N_1	27.2	-	-	82.6	79.4	64.0	76.0	78.2	64.6	50.6	60.9	61.7	66.7	69.5	73.3	77.3	81.7
WGS_NC_T_1	WGS_NC_N_1	29.6	-	-	83.0	84.8	70.3	80.0	79.6	71.7	66.0	72.1	73.4	77.3	78.0	81.6	84.8	87.6
WGS_NS_T_1	WGS_NS_N_1	29.9	-	-	81.1	83.9	71.2	69.8	77.1	73.3	70.2	75.8	79.8	81.7	81.2	82.0	86.5	88.3
WGS_NS_T_2	WGS_NS_N_2	29.6	-	-	80.9	83.2	69.6	73.2	75.9	69.7	65.6	71.1	67.2	72.3	77.5	80.9	82.0	86.0
WGS_NS_T_3	WGS_NS_N_3	26.6	-	-	80.2	83.7	68.7	71.0	77.2	69.0	65.6	72.8	72.6	77.2	77.5	80.6	83.6	86.4
WGS_NS_T_4	WGS_NS_N_4	28.7	-	-	81.6	84.0	71.2	70.1	77.4	71.2	68.0	75.0	79.0	81.1	81.1	82.6	86.0	89.3
WGS_NS_T_5	WGS_NS_N_5	29.3	-	-	79.4	82.8	69.6	67.4	71.5	71.1	67.3	72.9	72.1	77.3	78.1	82.2	83.6	87.0
WGS_NS_T_6	WGS_NS_N_6	30.8	-	-	81.4	84.0	71.1	67.9	74.0	73.9	70.0	76.9	79.6	82.0	81.3	83.7	87.4	89.7
WGS_NS_T_7	WGS_NS_N_7	30.1	-	-	80.3	84.2	71.7	66.5	71.6	72.0	68.1	75.0	77.6	80.8	81.3	83.4	86.8	89.2
WGS_NS_T_8	WGS_NS_N_8	30.3	-	-	79.9	83.5	69.7	74.6	75.3	68.7	65.9	70.7	66.9	72.0	76.4	81.0	80.8	85.2
WGS_NS_T_9	WGS_NS_N_9	28.6	-	-	81.1	84.8	71.6	73.9	78.3	67.9	65.4	72.1	66.9	74.2	78.6	81.8	81.6	85.8
WGS_NV_T_1	WGS_NV_N_1	39.7	-	-	84.6	86.5	73.3	74.3	80.7	79.2	73.9	79.7	83.6	85.8	82.7	84.9	88.2	89.9
WGS_NV_T_2	WGS_NV_N_2	39.4	-	-	83.8	86.9	73.0	74.2	80.3	80.4	74.2	79.5	83.5	85.1	82.6	84.3	88.5	90.1
WGS_NV_T_3	WGS_NV_N_3	40.0	-	-	85.2	86.5	72.6	76.0	80.1	79.6	75.3	79.6	82.7	84.5	83.6	85.9	87.8	89.8
Average		31.5	-	-	81.7	84.2	70.0	73.7	77.7	72.8	67.7	74.2	75.5	79.0	79.3	81.9	85.2	87.9

Table S3 - F1-score (%) performance of different somatic mutation detection methods and network trained models on the tumor-normal titration data set

Tumor	Normal	Tool															
											NeuSomatic-S				NeuSomatic			
		VarDict	SomaticSniper	MuSE	DRAGEN	Octopus-RF	Octopus-hard	MuTect2	Lancet	Strelka2	DREAM3	SEQC-WGS-Spike	SEQC-WGS-GT-50	SEQC-WGS-GT50-SpikeWGS10	DREAM3	SEQC-WGS-Spike	SEQC-WGS-GT-50	SEQC-WGS-GT50-SpikeWGS10
**SNVs**																		
SPP_10x_100%T	SPP_10x_100%N	40.1	32.5	49.6	46.1	74.8	46.2	7.1	34.6	47.9	45.2	55.1	57.2	58.8	33.9	66.3	71.2	69.6
SPP_10x_75%T	SPP_10x_100%N	34.9	27.7	47.0	35.9	69.3	49.8	6.9	27.9	41.7	43.6	53.1	48.6	50.6	33.8	62.1	65.4	64.1
SPP_10x_50%T	SPP_10x_100%N	25.4	18.9	36.9	22.7	58.0	41.2	5.9	17.4	29.2	38.2	41.1	31.6	33.9	29.0	49.9	51.4	49.8
SPP_10x_20%T	SPP_10x_100%N	6.8	4.5	12.3	5.0	25.7	17.6	2.6	3.3	7.3	15.9	12.8	5.9	7.1	12.2	18.5	18.5	16.6
SPP_10x_10%T	SPP_10x_100%N	1.8	1.0	3.8	1.0	9.3	6.3	1.0	0.6	1.9	6.8	3.9	1.2	1.7	4.7	6.0	5.8	4.9
SPP_10x_5%T	SPP_10x_100%N	0.5	0.2	1.1	0.2	3.0	2.1	0.3	0.1	0.7	2.3	1.0	0.2	0.4	1.7	1.9	1.7	1.4
SPP_30x_100%T	SPP_30x_100%N	43.2	69.8	84.5	88.6	90.9	81.2	84.9	83.8	84.4	78.4	85.0	86.9	87.8	84.0	89.7	92.0	91.9
SPP_30x_75%T	SPP_30x_100%N	42.0	68.3	81.2	86.0	88.8	87.3	83.1	80.0	82.8	76.1	84.8	82.9	84.1	82.3	88.0	89.6	89.4
SPP_30x_50%T	SPP_30x_100%N	39.5	60.1	71.8	78.4	84.5	78.5	78.1	70.1	77.6	56.8	77.1	72.6	74.7	69.4	83.1	83.8	83.2
SPP_30x_20%T	SPP_30x_100%N	23.8	24.8	34.1	45.8	61.0	59.0	50.8	36.1	45.8	33.6	45.7	37.1	40.5	45.6	55.7	56.1	53.8
SPP_30x_10%T	SPP_30x_100%N	8.9	5.9	10.0	17.6	33.4	33.5	23.0	11.7	17.8	15.6	20.2	14.2	16.7	23.6	27.0	26.3	23.9
SPP_30x_5%T	SPP_30x_100%N	2.2	0.8	1.9	4.1	13.5	14.3	7.3	2.4	4.2	5.4	5.7	3.6	4.5	9.1	9.2	8.3	6.7
SPP_50x_100%T	SPP_50x_100%N	39.6	65.3	88.6	92.7	94.4	86.0	86.4	89.4	89.4	88.9	91.2	92.9	93.4	91.8	93.8	94.9	95.0
SPP_50x_75%T	SPP_50x_100%N	38.6	65.2	85.9	91.1	93.0	91.9	85.8	87.0	89.0	87.9	90.4	90.5	91.0	91.0	92.9	93.3	93.3
SPP_50x_50%T	SPP_50x_100%N	37.0	61.6	79.3	87.4	89.1	88.5	83.5	81.5	85.8	79.6	84.6	84.8	85.3	86.6	89.6	89.9	89.7
SPP_50x_20%T	SPP_50x_100%N	28.4	28.9	44.7	65.3	71.5	67.3	66.3	55.8	63.6	42.4	59.7	59.9	60.7	62.9	70.0	70.5	70.3
SPP_50x_10%T	SPP_50x_100%N	13.4	6.5	14.7	35.2	43.9	43.3	37.5	26.1	33.8	20.8	32.1	32.9	33.6	35.8	40.7	42.1	41.0
SPP_50x_5%T	SPP_50x_100%N	4.5	0.9	2.3	12.0	17.9	20.1	13.5	7.6	11.2	7.7	11.0	12.0	12.3	13.8	14.9	16.2	14.9
SPP_80x_100%T	SPP_80x_100%N	51.7	62.4	91.6	95.4	96.9	89.1	88.6	93.3	92.4	92.5	94.6	95.7	96.0	95.2	95.5	97.7	97.5
SPP_80x_75%T	SPP_80x_100%N	44.3	62.4	88.9	93.9	95.2	94.1	86.5	91.0	92.3	91.0	93.2	93.7	93.9	93.8	95.3	95.9	95.7
SPP_80x_50%T	SPP_80x_100%N	36.2	61.0	83.9	91.3	92.6	91.7	84.5	87.5	90.0	87.7	89.4	90.3	90.5	91.7	92.8	93.4	93.0
SPP_80x_20%T	SPP_80x_100%N	28.7	30.0	55.0	77.6	80.7	73.4	75.6	70.5	74.0	49.1	68.2	67.6	67.3	78.9	80.0	78.4	80.0
SPP_80x_10%T	SPP_80x_100%N	14.5	6.5	20.8	53.8	58.9	54.3	53.2	44.2	49.1	24.8	41.4	41.3	41.1	56.5	56.8	55.7	57.0
SPP_80x_5%T	SPP_80x_100%N	5.2	0.6	3.3	24.5	29.2	28.1	26.2	17.8	20.6	9.5	15.6	17.4	17.3	27.5	27.2	27.5	26.9
SPP_100x_100%T	SPP_100x_100%N	55.8	60.8	92.6	95.6	97.3	89.8	89.1	94.6	93.3	93.4	95.7	96.5	96.7	95.9	95.6	98.2	98.0
SPP_100x_75%T	SPP_100x_100%N	48.9	61.3	90.1	94.3	96.1	95.1	87.6	92.7	93.8	92.0	94.2	94.5	94.8	95.2	96.2	96.9	96.6
SPP_100x_50%T	SPP_100x_100%N	37.3	60.5	85.5	92.4	93.2	92.1	84.5	89.0	91.3	88.3	90.6	91.3	91.5	92.8	93.8	94.1	93.8
SPP_100x_20%T	SPP_100x_100%N	29.8	30.0	59.4	77.8	83.9	83.7	78.3	75.7	77.4	66.5	72.8	74.4	74.4	82.6	83.6	84.5	83.5
SPP_100x_10%T	SPP_100x_100%N	15.3	5.9	25.0	53.8	65.9	59.4	59.4	52.5	54.3	26.8	45.2	43.4	43.1	63.0	63.8	61.6	63.6
SPP_100x_5%T	SPP_100x_100%N	5.2	0.5	3.7	24.5	36.3	33.5	33.1	24.6	24.6	10.3	18.4	19.2	18.9	33.6	34.0	33.5	33.6
SPP_200x_100%T	SPP_200x_100%N	67.3	57.5	95.0	97.2	98.0	91.4	92.4	96.2	94.9	94.2	97.5	97.7	97.8	97.0	94.8	98.8	98.8
SPP_200x_75%T	SPP_200x_100%N	60.0	57.1	92.5	96.5	97.2	96.9	91.1	95.2	96.2	92.4	96.5	96.0	96.1	97.0	97.4	97.9	97.5
SPP_200x_50%T	SPP_200x_100%N	50.9	55.8	88.7	95.0	95.9	95.5	87.5	93.8	94.7	89.2	94.4	93.5	93.5	95.7	96.6	96.4	95.6
SPP_200x_20%T	SPP_200x_100%N	36.1	22.9	72.0	87.9	89.5	90.4	82.4	85.7	85.6	73.4	84.6	84.0	83.7	88.4	90.7	89.6	87.4
SPP_200x_10%T	SPP_200x_100%N	20.4	2.4	40.4	74.6	78.7	81.6	70.3	74.8	73.4	48.2	67.6	66.7	66.6	75.9	80.1	77.3	72.7
SPP_200x_5%T	SPP_200x_100%N	5.6	0.1	8.3	46.4	56.1	55.1	51.9	54.6	50.5	17.9	36.4	34.3	34.0	50.7	58.8	51.9	43.9
SPP_300x_100%T	SPP_300x_100%N	73.1	56.4	96.7	97.3	97.9	91.7	93.3	96.3	95.1	93.9	97.7	98.1	98.3	97.0	94.5	98.9	98.9
SPP_300x_75%T	SPP_300x_100%N	68.7	57.6	93.8	95.8	97.5	97.4	92.9	95.9	96.9	91.8	97.2	96.9	97.0	97.5	97.6	98.6	98.3
SPP_300x_50%T	SPP_300x_100%N	55.8	55.5	90.2	95.9	96.4	96.2	82.2	95.0	95.9	88.4	95.4	95.0	95.0	96.6	97.3	97.6	97.0
SPP_300x_20%T	SPP_300x_100%N	39.5	18.4	76.8	88.9	90.3	92.1	79.4	89.8	87.8	71.1	86.3	86.4	85.6	91.2	93.1	91.6	89.6
SPP_300x_10%T	SPP_300x_100%N	20.7	1.3	46.9	77.6	81.2	85.7	69.6	79.3	77.7	42.5	70.9	70.4	69.4	81.2	86.0	81.7	77.3
SPP_300x_5%T	SPP_300x_100%N	5.1	0.0	13.2	52.6	63.4	62.7	54.2	66.4	60.6	14.5	43.5	43.2	41.4	61.9	72.1	62.9	54.0
SPP_80x_100%T	SPP_80x_95%N	46.0	62.8	59.5	80.0	85.6	80.7	43.8	47.8	90.9	89.8	93.5	94.7	94.9	91.5	95.0	97.0	96.3
SPP_80x_75%T	SPP_80x_95%N	38.6	61.3	52.3	71.7	83.6	83.3	41.2	44.5	89.1	87.7	91.5	92.0	92.3	89.2	93.6	94.8	93.8
SPP_80x_50%T	SPP_80x_95%N	30.9	58.6	39.8	56.8	79.8	80.3	37.8	39.2	82.8	82.8	86.1	86.9	87.0	85.7	89.0	91.0	89.3
SPP_80x_20%T	SPP_80x_95%N	23.1	25.7	12.2	26.6	62.0	60.2	26.5	23.6	45.2	37.7	55.1	54.9	55.2	60.2	61.5	67.8	63.3
SPP_80x_10%T	SPP_80x_95%N	10.7	4.7	2.2	12.1	35.9	40.0	13.6	10.6	17.9	13.3	23.8	24.9	25.3	32.4	29.3	37.7	31.1
Average		31.0	33.7	51.7	62.6	71.0	67.7	57.0	58.2	63.9	55.4	63.7	62.9	63.3	66.1	70.2	70.8	69.4
**INDELs**																		
SPP_10x_100%T	SPP_10x_100%N	15.0	-	-	42.5	58.8	31.5	13.0	33.2	35.4	26.3	35.8	31.1	38.0	40.2	42.9	47.0	55.4
SPP_10x_75%T	SPP_10x_100%N	13.8	-	-	35.6	53.6	39.0	14.1	29.8	31.6	29.7	38.4	28.9	37.8	46.2	45.5	41.3	52.8
SPP_10x_50%T	SPP_10x_100%N	9.7	-	-	22.1	41.3	29.5	9.9	17.3	21.9	23.7	33.2	16.9	25.3	39.0	35.9	27.7	38.2
SPP_10x_20%T	SPP_10x_100%N	2.8	-	-	3.7	13.8	11.0	5.5	3.1	3.3	15.9	16.7	3.5	6.7	25.0	14.2	5.4	11.1
SPP_10x_10%T	SPP_10x_100%N	0.5	-	-	0.3	3.6	3.1	0.9	0.3	0.5	8.7	8.3	0.7	2.2	12.7	3.5	0.9	2.0
SPP_10x_5%T	SPP_10x_100%N	0.1	-	-	0.0	1.2	0.9	0.6	0.0	0.1	4.4	4.8	0.3	1.0	5.6	1.5	0.1	0.4
SPP_30x_100%T	SPP_30x_100%N	26.0	-	-	77.8	80.7	67.4	75.9	75.7	68.0	58.9	68.7	68.3	73.5	72.8	76.5	81.0	84.9
SPP_30x_75%T	SPP_30x_100%N	26.5	-	-	77.8	80.0	77.0	75.6	74.9	68.2	61.7	75.1	68.1	74.7	73.2	81.1	80.0	83.7
SPP_30x_50%T	SPP_30x_100%N	17.8	-	-	68.0	74.0	53.1	60.5	64.5	60.1	38.9	66.5	55.6	64.9	57.7	74.4	70.1	75.1
SPP_30x_20%T	SPP_30x_100%N	10.5	-	-	36.4	48.6	39.4	41.5	33.8	26.2	28.9	46.9	28.7	40.6	43.9	49.2	40.3	47.1
SPP_30x_10%T	SPP_30x_100%N	3.7	-	-	11.4	21.2	17.5	14.7	11.2	7.6	16.7	27.6	10.9	16.5	24.9	21.5	12.8	19.1
SPP_30x_5%T	SPP_30x_100%N	1.2	-	-	3.0	7.1	6.2	4.4	2.7	1.5	8.7	14.3	4.5	7.9	12.1	6.3	4.7	6.2
SPP_50x_100%T	SPP_50x_100%N	27.0	-	-	81.4	83.7	69.2	72.8	78.7	74.7	67.6	75.0	79.8	81.7	78.6	82.1	85.4	87.6
SPP_50x_75%T	SPP_50x_100%N	28.0	-	-	81.8	83.1	80.4	74.3	79.0	75.0	72.5	82.5	79.1	81.3	78.6	85.4	85.1	87.9
SPP_50x_50%T	SPP_50x_100%N	23.8	-	-	79.2	80.4	78.9	71.2	75.6	69.9	64.6	78.0	72.5	76.0	77.3	82.1	80.7	84.4
SPP_50x_20%T	SPP_50x_100%N	12.7	-	-	55.6	61.3	46.7	48.9	52.7	42.5	35.7	56.3	47.5	53.1	54.2	65.3	57.4	64.2
SPP_50x_10%T	SPP_50x_100%N	6.4	-	-	27.7	34.0	24.9	20.8	25.4	13.3	20.7	33.6	25.2	28.5	33.2	35.9	28.8	35.2
SPP_50x_5%T	SPP_50x_100%N	2.4	-	-	8.6	14.5	10.8	8.6	8.5	3.2	11.0	18.2	11.4	13.9	15.3	14.6	11.5	15.1
SPP_80x_100%T	SPP_80x_100%N	36.6	-	-	83.0	85.6	71.9	73.2	80.0	77.8	73.1	78.0	83.5	83.8	82.3	83.2	87.6	89.1
SPP_80x_75%T	SPP_80x_100%N	31.3	-	-	83.1	84.2	80.6	71.8	80.4	78.7	76.9	84.0	83.2	84.5	81.8	86.1	87.9	88.8
SPP_80x_50%T	SPP_80x_100%N	24.5	-	-	82.6	82.7	80.9	70.6	79.0	76.1	73.1	82.4	78.8	80.8	80.7	83.9	84.4	86.3
SPP_80x_20%T	SPP_80x_100%N	12.1	-	-	65.8	68.0	46.2	50.4	64.9	55.7	38.9	63.3	57.2	60.0	64.5	72.0	67.3	72.1
SPP_80x_10%T	SPP_80x_100%N	7.3	-	-	44.9	46.3	32.0	26.6	42.3	26.4	23.4	43.1	33.9	37.6	44.6	50.8	41.7	48.7
SPP_80x_5%T	SPP_80x_100%N	2.9	-	-	20.1	22.1	15.4	12.5	19.7	7.7	10.9	22.7	16.1	18.1	23.4	25.5	17.3	23.7
SPP_100x_100%T	SPP_100x_100%N	39.1	-	-	83.4	85.6	71.8	73.3	79.5	78.4	74.4	78.9	83.8	84.7	83.2	83.9	87.3	90.1
SPP_100x_75%T	SPP_100x_100%N	35.1	-	-	83.3	84.8	81.4	73.3	80.7	80.4	78.3	84.7	84.0	84.9	82.3	87.0	88.0	89.2
SPP_100x_50%T	SPP_100x_100%N	23.9	-	-	82.5	83.0	80.0	68.1	78.5	77.5	73.8	82.5	80.4	81.3	80.4	84.0	85.1	86.4
SPP_100x_20%T	SPP_100x_100%N	17.2	-	-	70.9	72.4	75.0	66.3	70.5	60.7	57.1	68.9	61.2	64.2	72.1	75.8	72.3	76.3
SPP_100x_10%T	SPP_100x_100%N	7.0	-	-	44.9	49.9	32.0	28.9	48.6	31.0	26.6	46.2	34.4	39.6	51.6	56.9	45.7	54.0
SPP_100x_5%T	SPP_100x_100%N	2.6	-	-	20.1	26.3	15.6	14.3	25.5	10.5	12.2	24.0	15.8	18.8	25.5	29.7	20.4	27.4
SPP_200x_100%T	SPP_200x_100%N	47.4	-	-	83.9	85.2	74.1	74.5	77.3	80.3	76.2	80.1	83.1	85.0	81.9	82.4	87.5	90.0
SPP_200x_75%T	SPP_200x_100%N	43.7	-	-	84.3	84.5	81.2	75.4	77.6	81.9	79.6	86.3	82.2	84.7	82.0	86.8	88.4	90.2
SPP_200x_50%T	SPP_200x_100%N	35.7	-	-	84.2	83.1	82.4	73.2	78.2	80.2	76.4	84.7	79.8	81.8	80.0	86.0	86.7	88.1
SPP_200x_20%T	SPP_200x_100%N	22.5	-	-	80.6	76.7	80.6	68.4	76.9	69.6	61.8	78.2	63.4	68.9	73.3	80.9	75.1	79.9
SPP_200x_10%T	SPP_200x_100%N	12.2	-	-	69.9	63.2	75.5	57.3	70.6	44.7	42.0	67.0	41.8	50.2	62.0	71.5	54.6	65.0
SPP_200x_5%T	SPP_200x_100%N	2.6	-	-	44.6	38.9	22.0	18.2	52.5	18.1	17.1	43.7	20.2	27.2	42.0	50.8	28.3	40.1
SPP_300x_100%T	SPP_300x_100%N	50.8	-	-	84.2	84.4	74.1	74.2	76.2	81.0	75.4	80.1	83.3	84.3	80.9	82.5	87.6	89.3
SPP_300x_75%T	SPP_300x_100%N	48.5	-	-	83.4	83.5	82.4	75.3	77.1	82.6	79.3	86.4	84.3	85.5	82.0	86.2	88.8	90.2
SPP_300x_50%T	SPP_300x_100%N	39.1	-	-	83.7	82.9	82.7	70.9	77.2	80.0	75.7	85.0	80.6	81.9	79.9	86.3	87.0	88.4
SPP_300x_20%T	SPP_300x_100%N	24.6	-	-	80.4	75.0	81.2	68.1	77.0	70.3	61.0	78.6	65.4	71.4	75.2	81.9	78.9	81.9
SPP_300x_10%T	SPP_300x_100%N	12.6	-	-	72.9	63.8	78.1	52.7	73.3	47.2	37.4	69.3	43.7	51.7	66.6	75.2	62.8	71.2
SPP_300x_5%T	SPP_300x_100%N	2.1	-	-	50.6	44.1	17.4	11.0	62.3	15.2	15.7	49.0	21.6	27.1	47.6	57.1	38.3	49.4
SPP_80x_100%T	SPP_80x_95%N	31.3	-	-	73.8	72.7	65.1	38.3	38.9	76.9	68.5	76.9	78.9	80.9	74.5	82.9	83.4	86.8
SPP_80x_75%T	SPP_80x_95%N	26.6	-	-	68.9	71.4	71.2	36.8	37.6	75.3	71.0	81.4	78.6	80.5	76.4	84.4	82.7	85.6
SPP_80x_50%T	SPP_80x_95%N	20.0	-	-	54.6	68.6	70.9	33.4	33.5	66.3	67.6	77.1	72.1	75.6	74.0	79.2	75.9	80.4
SPP_80x_20%T	SPP_80x_95%N	9.2	-	-	20.1	49.4	35.3	16.3	19.5	22.2	31.0	52.3	41.8	46.8	50.5	55.2	44.7	52.9
SPP_80x_10%T	SPP_80x_95%N	4.9	-	-	8.3	25.1	22.2	6.2	10.2	3.6	14.3	27.0	17.5	21.8	27.1	25.5	15.3	22.2
Average		19.2	-	-	55.4	59.2	52.5	44.5	51.7	48.1	46.0	58.3	50.7	54.6	57.8	61.5	57.7	62.4

Table S4 - F1-score (%) performance of different somatic mutation detection methods and network trained models on the library-preparation data set

		Tool																
											NeuSomatic-S				NeuSomatic			
Tumor	Normal	VarDict	SomaticSniper	MuSE	DRAGEN	Octopus-RF	Octopus-hard	MuTect2	Lancet	Strelka2	DREAM3	SEQC-WGS-Spike	SEQC-WGS-GT-50	SEQC-WGS-GT50-SpikeWGS10	DREAM3	SEQC-WGS-Spike	SEQC-WGS-GT-50	SEQC-WGS-GT50-SpikeWGS10
										SNVs								
LBP_Nextera_T_100ng	LBP_Nextera_N_100ng	47.4	66.3	89.0	92.6	94.8	86.6	89.8	88.7	88.9	88.6	89.7	91.8	92.5	93.0	93.7	95.3	95.1
LBP_Nextera_T_10ng	LBP_Nextera_N_10ng	46.8	62.5	90.0	93.1	94.9	86.8	89.7	90.3	89.2	90.1	91.8	93.4	93.7	93.1	93.8	95.5	95.4
LBP_Nextera_T_1ng	LBP_Nextera_N_1ng	37.1	63.1	86.4	89.9	91.8	72.8	86.0	85.6	82.4	85.4	88.5	90.6	91.3	89.2	90.3	92.3	92.3
LBP_TruSeq_T_100ng	LBP_TruSeq_N_100ng	39.5	67.5	86.3	90.5	92.8	84.1	86.5	86.3	85.9	86.6	87.2	89.2	89.8	90.4	91.7	93.1	93.0
LBP_TruSeq_T_10ng	LBP_TruSeq_N_10ng	40.1	68.6	82.2	86.4	84.6	48.6	80.3	80.8	79.7	75.4	81.5	85.5	85.8	79.6	84.8	88.3	89.0
LBP_TruSeq_T_1ng	LBP_TruSeq_N_1ng	30.2	20.2	23.1	30.5	50.5	0.0	3.7	18.8	34.0	16.6	17.3	25.6	23.1	19.2	51.3	50.2	47.9
Average		40.2	58.0	76.2	80.5	84.9	63.2	72.7	75.1	76.7	73.8	76.0	79.4	79.4	77.4	84.3	85.8	85.5
										INDELs								
LBP_Nextera_T_100ng	LBP_Nextera_N_100ng	26.8	-	-	83.3	80.1	60.2	61.3	80.3	69.7	68.1	71.1	74.9	77.0	79.9	77.4	82.4	80.4
LBP_Nextera_T_10ng	LBP_Nextera_N_10ng	18.0	-	-	83.2	74.2	41.9	38.6	80.2	69.6	69.1	67.9	76.0	75.1	80.2	69.3	73.7	65.9
LBP_Nextera_T_1ng	LBP_Nextera_N_1ng	9.9	-	-	79.3	63.1	19.7	37.4	76.2	48.0	60.3	59.8	68.4	66.4	76.6	60.2	69.2	55.5
LBP_TruSeq_T_100ng	LBP_TruSeq_N_100ng	15.6	-	-	82.3	75.0	47.9	56.1	78.1	65.1	62.9	67.2	72.1	73.9	77.6	71.2	79.0	72.8
LBP_TruSeq_T_10ng	LBP_TruSeq_N_10ng	12.7	-	-	75.1	68.0	16.4	52.3	72.7	52.7	51.2	54.7	65.8	66.4	70.8	63.6	74.2	69.3
LBP_TruSeq_T_1ng	LBP_TruSeq_N_1ng	9.5	-	-	31.5	37.2	0.2	7.7	17.0	20.8	15.0	12.6	26.0	23.4	23.5	21.3	25.8	37.5
Average		15.4	-	-	72.5	66.3	31.1	42.2	67.4	54.3	54.4	55.6	63.9	63.7	68.1	60.5	67.4	63.6

Table S5 - F1-score (%) performance of different somatic mutation detection methods and network trained models on the WES data set

		Tool																						
											NeuSomatic-S							NeuSomatic						
Tumor	Normal	VarDict	SomaticSniper	MuSE	DRAGEN	Octopus-RF	Octopus-hard	MuTect2	Lancet	Strelka2	DREAM3	WES-TCGA	SEQC-WGS-Spike	SEQC-WGS-GT-50	SEQC-WGS-GT50-SpikeWGS10	SEQC-WES-Spike	SEQC-WES-SpikeWGS10	DREAM3	WES-TCGA	SEQC-WGS-Spike	SEQC-WGS-GT-50	SEQC-WGS-GT50-SpikeWGS10	SEQC-WES-Spike	SEQC-WES-SpikeWGS10
												SNVs												
WES_EA_T_1	WES_EA_N_1	84.1	75.6	89.9	93.8	95.2	88.0	93.9	91.7	90.2	90.7	91.7	90.9	92.3	92.7	89.6	87.8	95.2	95.1	91.7	95.5	95.6	95.5	94.9
WES_FD_T_1	WES_FD_N_1	69.0	73.9	82.7	83.2	89.5	81.7	86.6	83.5	79.4	83.1	81.2	81.2	83.6	83.9	79.4	67.2	90.8	90.1	81.8	91.1	90.7	90.5	88.2
WES_FD_T_2	WES_FD_N_2	74.9	75.9	81.8	85.3	88.8	80.1	86.9	85.0	82.8	85.6	80.3	83.6	85.7	86.0	80.6	70.4	90.2	91.0	86.1	91.8	91.2	91.0	88.2
WES_FD_T_3	WES_FD_N_3	72.1	72.8	81.9	84.6	87.8	79.1	86.6	83.0	79.6	83.0	79.5	80.5	82.3	82.6	78.8	68.9	89.9	89.9	84.0	90.4	90.3	89.5	87.8
WES_IL_T_1	WES_IL_N_1	79.4	74.5	91.2	95.4	94.2	79.2	94.8	94.1	91.9	90.1	93.4	92.3	93.6	93.5	89.8	88.4	97.4	96.1	94.6	97.2	96.5	97.0	96.6
WES_IL_T_2	WES_IL_N_2	92.5	84.0	93.8	98.8	91.3	79.0	96.8	88.6	97.3	94.2	96.2	97.8	97.9	98.1	94.3	95.6	97.6	98.3	97.8	99.2	98.9	99.0	98.7
WES_IL_T_3	WES_IL_N_3	88.9	77.9	94.2	97.4	92.7	84.0	96.4	95.2	94.2	94.9	95.1	96.0	97.7	97.9	91.2	89.0	97.2	97.3	96.0	98.9	98.5	98.5	97.6
WES_LL_T_1	WES_LL_N_1	66.7	71.3	78.3	83.1	87.3	77.9	84.1	81.0	77.8	79.9	78.6	77.1	80.0	80.6	76.1	63.8	87.7	88.6	81.9	87.1	87.7	87.3	85.6
WES_NC_T_1	WES_NC_N_1	79.7	76.1	87.4	91.0	92.1	82.3	91.7	89.2	87.2	90.6	87.7	87.8	90.3	91.3	87.8	82.1	94.6	93.7	90.3	94.3	94.4	93.9	91.9
WES_NV_T_1	WES_NV_N_1	93.2	79.7	95.2	98.9	90.0	80.7	97.6	97.4	95.6	95.9	97.1	97.1	97.8	97.5	94.9	95.3	98.3	98.4	97.1	99.2	98.7	98.6	98.2
WES_NV_T_2	WES_NV_N_2	91.4	79.9	94.8	98.7	88.6	80.2	97.8	95.5	95.8	94.8	97.0	97.3	97.9	98.1	96.0	95.2	98.4	98.2	97.1	99.1	98.6	98.5	98.2
WES_NV_T_3	WES_NV_N_3	91.7	79.3	94.2	98.8	89.1	82.6	97.9	96.8	95.7	94.5	96.4	97.2	97.4	97.8	94.9	94.2	98.7	98.2	97.2	99.2	98.7	98.9	98.5
Average		82.0	76.7	88.8	92.4	90.6	81.2	92.6	90.1	89.0	89.8	89.5	89.9	91.4	91.7	87.8	83.2	94.7	94.6	91.3	95.3	95.0	94.9	93.7

Table S6 - F1-score (%) performance of different somatic mutation detection methods and network trained models on the AmpliSeq data set

		Tool																						
											NeuSomatic-S							NeuSomatic						
Tumor	Normal	VarDict	SomaticSniper	MuSE	DRAGEN	Octopus-RF	Octopus-hard	MuTect2	Lancet	Strelka2	DREAM3	WES-TCGA	SEQC-WGS-Spike	SEQC-WGS-GT-50	SEQC-WGS-GT50-SpikeWGS10	SEQC-WES-Spike	SEQC-WES-SpikeWGS10	DREAM3	WES-TCGA	SEQC-WGS-Spike	SEQC-WGS-GT-50	SEQC-WGS-GT50-SpikeWGS10	SEQC-WES-Spike	SEQC-WES-SpikeWGS10
												SNVs												
AmpliSeq_T_1	AmpliSeq_T_1	80.9	72.0	78.6	88.0	47.3	0.0	77.8	67.4	89.6	68.3	86.9	77.1	82.3	82.7	81.6	82.7	61.7	90.1	90.6	90.1	87.8	91.4	89.7
AmpliSeq_T_2	AmpliSeq_T_2	79.9	69.4	80.1	88.4	47.3	0.0	77.7	64.7	89.3	64.0	83.5	76.0	81.2	80.9	82.0	81.1	56.9	88.2	89.1	88.8	85.8	89.9	90.5
AmpliSeq_T_3	AmpliSeq_T_3	81.1	71.8	78.7	87.1	50.6	0.0	78.2	67.6	90.2	65.5	86.2	76.8	81.0	83.2	83.2	83.5	57.2	88.7	89.2	91.3	86.7	91.2	90.6
Average		80.6	71.1	79.1	87.8	48.4	0.0	77.9	66.6	89.7	65.9	85.5	76.6	81.5	82.3	82.3	82.4	58.6	89.0	89.6	90.1	86.8	90.8	90.3

Table S7 - F1-score (%) performance of different somatic mutation detection methods and network trained models on the FFPE WGS data set

		Tool																				
											NeuSomatic-S						NeuSomatic					
Tumor	Normal	VarDict	SomaticSniper	MuSE	DRAGEN	Octopus-RF	Octopus-hard	MuTect2	Lancet	Strelka2	DREAM3	SEQC-WGS-Spike	SEQC-WGS-GT-50	SEQC-WGS-GT50-SpikeWGS10	SEQC-FFPE-Spike	SEQC-FFPE-SpikeWGS10	DREAM3	SEQC-WGS-Spike	SEQC-WGS-GT-50	SEQC-WGS-GT50-SpikeWGS10	SEQC-FFPE-Spike	SEQC-FFPE-SpikeWGS10
SNVs																						
FFG_GZ_T_1h-A	FFG_GZ_N_1h-B	34.5	62.3	81.1	83.2	84.5	74.6	74.1	75.8	80.1	80.0	81.7	85.0	84.9	77.8	77.4	81.6	84.7	85.3	85.9	82.4	79.8
FFG_GZ_T_1h-A	WGS_IL_N_1	32.3	57.8	82.6	79.3	82.2	71.0	77.9	80.4	76.5	77.4	80.7	84.1	83.4	79.3	78.1	78.6	83.9	83.7	85.6	82.9	79.9
FFG_GZ_T_24h-B	FFG_GZ_N_24h-B	43.6	64.6	83.9	83.4	84.8	75.1	74.7	75.8	80.7	81.0	82.9	85.5	85.3	79.1	79.0	82.2	84.8	85.5	86.4	83.2	80.8
FFG_GZ_T_24h-B	WGS_IL_N_1	35.9	58.4	83.1	79.5	83.1	73.0	78.5	81.0	77.2	79.3	81.8	84.7	84.2	79.8	78.8	81.4	84.1	84.5	86.1	83.4	80.7
FFG_GZ_T_2h-A	FFG_GZ_N_2h-A	46.1	63.6	82.1	84.0	84.6	77.7	71.3	71.6	80.9	80.7	83.2	85.3	85.3	78.2	78.6	81.7	85.1	85.9	86.2	82.7	81.7
FFG_GZ_T_2h-A	WGS_IL_N_1	37.9	57.6	83.3	78.9	81.6	73.6	79.6	80.8	75.4	78.2	80.8	83.9	83.3	79.5	78.4	79.7	84.0	83.8	85.8	83.8	81.9
FFG_GZ_T_6h-A	FFG_GZ_N_6h-A	41.9	63.9	83.4	83.6	84.6	76.0	74.4	73.8	78.9	78.8	82.5	84.9	84.7	77.8	78.0	79.6	84.5	84.9	86.0	82.2	80.5
FFG_GZ_T_6h-A	WGS_IL_N_1	35.0	57.8	83.6	79.6	82.8	73.7	79.7	80.2	75.2	76.9	81.0	84.1	83.6	79.0	78.0	78.3	83.9	83.8	85.6	82.9	80.3
FFG_IL_T_1h	FFG_IL_N_1h	39.3	62.1	79.2	83.0	84.8	76.3	59.8	71.7	79.9	80.8	83.8	85.5	85.6	76.6	81.2	83.7	84.4	86.3	86.4	80.2	78.8
FFG_IL_T_1h	WGS_IL_N_1	36.5	52.6	83.1	82.7	85.6	76.2	67.1	83.3	77.0	81.3	84.0	85.6	85.6	81.1	82.2	82.0	84.4	86.3	86.7	83.4	79.7
FFG_IL_T_24h	FFG_IL_N_24h	33.1	61.7	73.5	80.7	83.4	76.8	58.3	63.1	79.3	80.3	82.5	84.8	84.7	71.1	78.8	81.6	84.6	85.4	85.7	78.9	80.1
FFG_IL_T_24h	WGS_IL_N_1	29.7	49.8	80.9	81.9	85.2	76.6	68.1	82.1	75.0	79.6	82.4	84.9	84.9	78.8	80.6	77.1	84.0	85.0	85.9	83.9	81.6
FFG_IL_T_2h	FFG_IL_N_2h	42.1	61.9	84.2	84.8	85.2	78.0	77.6	82.0	79.3	81.5	83.1	85.1	85.1	79.8	81.4	83.0	84.7	85.7	86.1	83.2	80.5
FFG_IL_T_2h	WGS_IL_N_1	35.5	51.3	82.2	81.5	84.8	76.4	70.1	82.0	74.3	79.6	82.5	84.7	84.7	79.7	81.2	78.5	83.7	84.9	85.7	80.9	80.1
FFG_IL_T_6h	FFG_IL_N_6h	43.6	63.4	83.3	84.9	85.5	78.9	77.2	83.1	80.3	81.4	83.6	85.6	85.6	80.7	81.9	85.4	84.1	86.4	86.6	84.4	81.4
FFG_IL_T_6h	WGS_IL_N_1	42.2	54.1	83.9	82.8	85.7	78.4	74.5	83.4	78.8	81.6	84.0	85.8	85.7	81.2	82.3	84.3	84.5	86.5	86.8	84.5	81.4
Average		38.1	58.9	82.1	82.1	84.3	75.8	72.7	78.1	78.1	79.9	82.5	85.0	84.8	78.7	79.7	81.2	84.3	85.2	86.1	82.7	80.6
INDELs																						
FFG_GZ_T_1h-A	FFG_GZ_N_1h-B	24.9	-	-	77.4	76.2	58.8	43.5	68.6	67.4	64.7	66.4	70.4	72.6	62.4	66.2	73.0	74.8	76.6	78.7	77.2	77.5
FFG_GZ_T_1h-A	WGS_IL_N_1	21.8	-	-	69.0	70.3	39.2	24.2	73.8	61.6	61.6	62.4	71.1	69.9	64.4	66.6	68.3	63.4	75.7	73.9	78.8	77.9
FFG_GZ_T_24h-B	FFG_GZ_N_24h-B	31.7	-	-	77.2	76.6	58.5	41.1	68.9	72.4	66.4	68.6	73.1	74.5	63.4	66.8	72.9	74.3	77.1	79.2	77.2	77.9
FFG_GZ_T_24h-B	WGS_IL_N_1	24.8	-	-	69.3	72.6	38.9	23.4	74.0	64.0	63.0	64.2	72.8	71.8	65.6	66.5	70.2	63.8	76.3	76.2	78.5	78.0
FFG_GZ_T_2h-A	FFG_GZ_N_2h-A	35.3	-	-	79.1	76.7	64.0	46.8	66.1	73.4	67.4	68.8	70.3	72.5	62.3	65.8	74.8	74.9	76.3	78.6	77.2	78.4
FFG_GZ_T_2h-A	WGS_IL_N_1	26.4	-	-	68.8	69.6	43.5	27.8	73.9	62.8	64.1	63.6	71.8	71.0	65.1	66.9	69.8	66.4	75.6	76.3	78.7	78.3
FFG_GZ_T_6h-A	FFG_GZ_N_6h-A	27.3	-	-	77.7	76.0	58.9	41.3	67.8	70.7	63.4	65.8	70.5	70.3	61.7	65.7	71.8	72.9	76.4	76.5	76.4	77.7
FFG_GZ_T_6h-A	WGS_IL_N_1	21.6	-	-	70.0	72.5	40.6	25.4	73.8	60.0	60.2	61.5	70.2	69.8	64.3	65.4	68.1	63.0	75.5	73.3	78.5	78.2
FFG_IL_T_1h	FFG_IL_N_1h	25.2	-	-	76.2	74.3	58.7	43.2	64.0	67.6	62.8	67.1	73.8	74.0	61.5	66.8	72.0	71.8	76.7	78.4	76.0	76.6
FFG_IL_T_1h	WGS_IL_N_1	24.1	-	-	76.3	73.4	52.0	39.9	73.7	64.8	62.1	64.1	74.0	74.1	65.6	67.7	73.5	72.2	77.5	77.4	78.9	77.8
FFG_IL_T_24h	FFG_IL_N_24h	26.0	-	-	75.9	73.9	65.4	48.3	56.0	67.8	62.8	66.9	71.8	73.4	55.1	64.8	73.0	74.3	76.5	77.3	72.4	76.3
FFG_IL_T_24h	WGS_IL_N_1	23.9	-	-	76.6	75.3	60.5	51.3	73.4	63.5	60.1	63.1	73.0	73.2	64.1	66.0	72.7	72.4	77.1	75.9	77.8	77.4
FFG_IL_T_2h	FFG_IL_N_2h	27.5	-	-	78.1	76.0	63.0	55.1	73.5	68.1	64.2	66.6	73.6	73.8	63.8	67.5	73.8	72.9	76.8	76.9	76.3	77.3
FFG_IL_T_2h	WGS_IL_N_1	23.2	-	-	75.5	73.1	55.5	42.9	72.9	62.3	60.0	62.4	73.5	71.9	63.8	66.4	72.9	70.4	76.3	74.8	77.2	77.0
FFG_IL_T_6h	FFG_IL_N_6h	26.6	-	-	77.2	74.5	61.6	56.3	75.7	65.8	62.0	66.3	73.8	73.7	65.0	67.4	73.0	69.9	77.0	78.7	78.2	78.1
FFG_IL_T_6h	WGS_IL_N_1	25.8	-	-	76.6	73.5	55.7	45.3	74.3	66.1	61.8	64.8	74.5	73.9	65.9	68.0	74.9	72.5	77.1	78.9	78.3	78.2
Average		26.0	-	-	75.1	74.0	54.7	41.0	70.7	66.1	62.9	65.2	72.4	72.5	63.4	66.5	72.2	70.6	76.5	76.9	77.4	77.7

Table S8 - F1-score (%) performance of different somatic mutation detection methods and network trained models on the FFPE WES data set

		Tool																														
											NeuSomatic-S											NeuSomatic										
Tumor	Normal	VarDict	SomaticSniper	MuSE	DRAGEN	Octopus-RF	Octopus-hard	MuTect2	Lancet	Strelka2	DREAM3	SEQC-WGS-Spike	SEQC-WGS-GT-50	SEQC-WGS-GT50-SpikeWGS10	SEQC-FFPE-Spike	SEQC-FFPE-SpikeWGS10	WES-TCGA	SEQC-WES-Spike	SEQC-WES-SpikeWGS10	SEQC-FFPE-WES-Spike	SEQC-FFPE-WES-SpikeWGS10	DREAM3	SEQC-WGS-Spike	SEQC-WGS-GT-50	SEQC-WGS-GT50-SpikeWGS10	SEQC-FFPE-Spike	SEQC-FFPE-SpikeWGS10	WES-TCGA	SEQC-WES-Spike	SEQC-WES-SpikeWGS10	SEQC-FFPE-WES-Spike	SEQC-FFPE-WES-SpikeWGS10
SNVs																																
FFX_GZ_T_1h_1	FFX_GZ_N_1h_1	58.0	57.1	65.8	67.7	68.9	59.5	65.5	58.9	63.1	67.0	65.7	64.3	63.2	61.2	64.1	66.0	63.5	61.3	62.9	58.7	69.1	68.7	69.7	69.3	61.1	59.4	68.3	70.4	67.8	69.1	68.1
FFX_GZ_T_1h_1	WES_IL_N_1	60.2	58.5	66.6	68.5	69.2	59.7	68.6	61.9	64.3	67.2	64.8	53.0	49.9	62.7	63.1	66.7	65.8	62.3	62.5	57.2	70.5	69.5	66.6	67.5	62.9	60.5	70.0	69.6	69.2	69.8	68.9
FFX_GZ_T_24h_1	FFX_GZ_N_24h_1	59.9	60.0	69.2	69.7	70.8	61.1	65.4	67.2	65.3	69.5	68.0	70.4	70.6	64.6	66.9	68.1	66.0	62.0	65.9	62.8	70.2	68.8	71.8	71.5	62.9	61.1	71.7	72.2	70.8	71.5	70.9
FFX_GZ_T_24h_1	WES_IL_N_1	64.8	61.1	71.4	71.4	71.8	63.7	72.5	69.2	69.0	69.7	69.3	65.2	63.3	66.1	67.6	70.7	69.3	68.4	65.2	60.6	73.4	72.4	72.6	72.1	65.1	63.5	72.8	73.3	72.9	72.3	72.5
FFX_GZ_T_6h_3	FFX_GZ_N_6h_1	58.2	60.3	68.9	69.5	71.2	61.2	66.9	65.5	63.3	69.0	67.8	69.7	69.2	64.6	65.2	66.5	64.5	62.9	64.3	60.2	70.0	69.8	71.6	71.4	63.7	62.0	71.4	71.7	69.8	71.2	70.3
FFX_GZ_T_6h_3	WES_IL_N_1	62.1	62.5	70.0	70.9	71.4	63.3	71.6	66.7	67.1	69.1	68.0	62.0	60.0	65.0	64.8	69.6	67.2	66.4	65.1	60.2	72.1	72.4	70.5	70.8	65.5	63.8	72.5	71.9	71.7	72.0	72.2
FFX_IL_T_1h_1	FFX_IL_N_1h_2	80.8	75.1	81.7	88.6	81.2	59.8	81.2	70.8	85.9	87.9	87.7	88.3	87.7	81.8	86.8	87.8	84.1	88.1	80.7	76.4	89.2	89.3	89.5	89.4	83.4	81.8	88.5	89.0	88.3	86.2	87.6
FFX_IL_T_1h_1	WES_IL_N_1	79.4	72.5	86.4	87.1	82.4	58.3	84.3	87.1	81.6	86.9	86.7	87.7	88.1	83.2	85.5	85.1	83.9	85.8	80.4	77.3	86.4	85.5	87.4	87.8	81.3	79.0	86.7	88.1	87.8	86.5	87.0
FFX_IL_T_24h_1	FFX_IL_N_24h_2	69.5	71.9	20.1	66.7	35.6	21.1	15.8	12.9	83.6	84.0	86.2	83.5	82.6	53.5	79.5	76.9	76.0	83.6	71.0	57.9	72.3	83.2	83.9	79.1	60.9	64.8	84.5	81.3	81.5	80.7	74.6
FFX_IL_T_24h_1	WES_IL_N_1	80.6	73.3	86.4	87.5	83.7	45.9	84.9	87.1	83.4	86.9	86.8	87.1	87.1	80.7	84.6	86.1	84.8	85.1	79.6	74.9	86.4	86.9	88.1	88.1	80.1	77.3	87.5	88.3	88.1	86.7	87.0
FFX_IL_T_2h_1	FFX_IL_N_2h_2	69.2	67.1	71.7	75.9	76.4	20.4	62.2	64.9	74.4	79.5	75.6	72.8	72.3	65.7	76.1	78.2	77.0	80.8	70.9	61.2	72.1	74.2	79.7	77.0	65.9	62.4	78.7	82.6	83.0	79.5	72.4
FFX_IL_T_2h_1	WES_IL_N_1	77.6	72.3	84.8	86.1	83.7	21.5	84.1	84.9	81.5	84.9	84.3	83.8	83.7	74.8	81.6	84.3	82.3	82.8	76.8	65.4	86.1	85.6	87.2	86.6	78.9	75.4	86.4	86.2	86.5	84.6	84.9
FFX_IL_T_6h_1	FFX_IL_N_6h_2	80.4	70.8	76.0	87.8	78.2	73.6	66.1	61.4	83.8	86.0	86.9	81.5	79.5	80.8	86.0	85.6	83.1	87.3	76.3	69.1	87.0	88.3	86.5	85.7	82.5	82.3	88.4	88.7	87.6	86.8	84.8
FFX_IL_T_6h_1	WES_IL_N_1	78.9	72.1	86.4	87.5	83.3	76.6	84.5	87.2	80.0	85.8	85.6	87.3	87.2	83.9	84.6	85.0	83.2	84.9	80.1	74.5	87.6	85.6	87.8	88.2	85.7	83.4	86.5	88.2	88.4	87.3	87.6
Average		70.0	66.8	71.8	77.5	73.4	53.3	69.5	67.6	74.7	78.1	77.4	75.5	74.6	70.6	75.5	76.9	75.1	75.8	71.6	65.5	78.0	78.6	79.5	78.9	71.4	69.8	79.6	80.1	79.5	78.9	77.8

Table S9 - F1-score (%) performance for the SEQC-WGS-Spike model and two sample-specific network trained models

Sample	NeuSomatic-S			NeuSomatic		
	SEQC-WGS-Spike	Sample	Sample+10%WGS	SEQC-WGS-Spike	Sample	Sample+10%WGS
**SNVs**						
WGS-NovaSeq	89.9	91.4	90.7	94.2	94.7	94.4
Titration-50%T-30X	77.1	74.9	75.4	83.1	82.7	82.0
Titration-10%T-100X	45.2	46.0	46.1	63.8	64.4	62.3
Library-prep-1ng-Nextera	88.5	86.3	86.9	90.3	91.9	91.4
WGS-FFPE-24h (FFPE-normal)	82.9	83.7	81.2	84.8	86.0	85.8
WGS-FFPE-24h (Fresh-normal)	81.8	80.1	80.8	84.1	85.9	86.2
WES	97.1	82.7	97.6	97.1	96.4	97.5
WES-FFPE-24h (FFPE-normal)	86.2	53.3	84.9	83.2	82.7	87.0
WES-FFPE-24h (Fresh-normal)	86.8	54.9	82.3	86.9	86.3	86.6
Average	81.7	72.6	80.7	85.3	85.7	85.9
**INDELs**						
WGS-NovaSeq	75.8	78.5	77.3	82.0	81.8	85.3
Titration-50%T-30X	66.5	65.0	68.0	74.4	74.2	67.6
Titration-10%T-100X	46.2	43.1	45.6	56.9	54.2	56.3
Library-prep-1ng-Nextera	59.8	67.3	67.1	60.2	79.6	78.1
WGS-FFPE-24h (FFPE-normal)	68.6	66.1	66.9	74.3	78.4	77.6
WGS-FFPE-24h (Fresh-normal)	64.2	65.7	64.3	63.8	78.8	78.6
Average	63.5	64.3	64.9	68.6	74.5	73.9

Table S10 - Computational complexity comparison of different somatic mutation detection algorithms. CPU time (hours) and maximum memory (GB) are shown for NA12878.BRCA sample with 220x tumor coverage and 75x normal coverage

**Tool**	**CPU time (hours)**	**Maximum Memory (GB)**
NeuSomatic	785	44
NeuSomatic-S	153	44
Strelka2	55	1
Lancet	5014	85
MuTect2	168	5
Octopus-hard	7150	24
Octopus-RF	7500	24
MuSE	170	0
SomaticSniper	16	7
VarDict	161	3

* DRAGEN spent 1.4h on FPGA to finish for this sample. Since DRAGEN ran on FPGA and does not use memory the same way as a CPU the CPU time and maximum memory usage are not comparable with other techniques.
