# Supplementary material for: Achieving robust somatic mutation detection with deep learning models derived from reference data sets of a cancer sample
Source: Genome Biol. 2022 Jan 7;23:12. doi: 10.1186/s13059-021-02592-9 (PMC8740374; doi:10.1186/s13059-021-02592-9)
Supplement: Supplementary file 3 — Additional file 3:. Review history [file 13059_2021_2592_MOESM3_ESM.docx]

Review History

**First round of review**

**Reviewer 1**

**Are you able to assess all statistics in the manuscript, including the appropriateness of statistical tests used?**

There are no statistics in the manuscript.

**Comments to author:**

Sahraeian et al. performed a comprehensive comparison of multiple somatic mutation detection methods in the well-characterized somatic reference datasets of the SEQC-II consortium, with considering the influence of sequencing depth, tissue type and purity. The manuscript underline their former developed deep learning framework, called NeuSomatic, was best somatic mutation caller. Somatic mutation detection is very import for cancer/disease study. The Sanger, DKFZ, Broad institute and other group did a broad study on how different somatic mutation caller performed in the ICGC and the TCGA's whole genome sequencing data (The ICGC/TCGA Pan-Cancer Analysis of Whole Genomes Consortium, Nature 2020; Alioto, et al. Nat Commun 2015; Pei et al, Briefings in Bioinformatics 2021). In title and abstract, the author emphasis that "Deep Learning Models" was used for robust somatic mutation detection, but only one deep learning method, NeuSomatic, was used in their study. Another small problems, the author said the NeuSomatic was first deep learning framework for somatic mutation caller, but as my poor knowledge, DeepVariant (Poplin et al., Nat. Biotech 2018) was the first one, which was showed in the NeuSomatic paper.

In general, Sahraeian et al.'s work did a comprehensive comparison on somatic mutation discovery in cancer genomics data, but some similar work already published. My suggestion is the manuscript not suit for publishing in Genome Biology.

My suggestion is the manuscript with following major revision:

1. Added DeepVariant and VarScan2 to the comparison.

2. Test the power of deep learning model in ICGC/TCGA's WGS/WES data at least three cancer type.

3. Time the computational time and how many computer resource costed by each method.

4. Literature review was limited in SEQC-II project, other similar work should be cited, both genomic data and method.

**Reviewer 2**

**Are you able to assess all statistics in the manuscript, including the appropriateness of statistical tests used?**

There are no statistics in the manuscript.

**Comments to author:**

This manuscript assesses the accuracy of several computational methods, including a deep-learning approach, for calling somatic mutations from short-read sequencing data. A number of different training strategies are evaluated and accuracy is assessed via a set of higher confidence mutations from a held out region of HCC1395. Using the SEQC-II data set, performance is compared across a several of different sequencing settings (WGS, WES, fresh, FFPE, etc.)

Overall, this is a comprehensive study that describes training-based improvements to a previously developed DL method, resulting in higher accuracy. It is likely to be of interest to those working in the important field of somatic mutation detection and to those developing predictive methods for related problems.

Comments:

1. The text is vague and imprecise in several places to such a degree that it becomes difficult to determine what was done. For example:

a- the definition of TP and FP, FN, etc. should be more precise: must the indel be predicted exactly? Presumably the SNV base must also be called correctly. These should be stated directly.

b- too terse to be understandable: line 151 "Through tumor-normal titration of WGS samples,'

c- the experimental set up described in the "Effect of FFPE processing" section is not clear. (line 181-187)

d- line 241: "private false negative calls" is not defined.

e- Section "Training data sets and models", the word "identified" is used repeatedly in this section, but it is not clear what that means. How were these candidate mutations identified? It's particularly not clear since in some cases these data sets were created by synthetically implanting mutations.

2. The main methodological innovation is the construction and evaluation of various strategies for training the NeuSomatic DL model. While the approaches are reasonable, the methodological innovation is moderate. More critically, it's not clear to what extent these strategies generalize to other DL or ML-based approaches. Are there other DL or ML-based approaches to which the same training sets could be applied to determine how specific to NeuSomatic the approaches are?

3. Links to source code for construction of the training data, and the ability to run the NeuSomatic predictor to reproduce results is needed. Ideally, source code to reproduce all the experiments in the paper should be provided.

**Authors Response**

**Point-by-point responses to the reviewers’ comments:**

Reviewer #1: Sahraeian et al. performed a comprehensive comparison of multiple somatic mutation detection methods in the well-characterized somatic reference datasets of the SEQC-II consortium, with considering the influence of sequencing depth, tissue type and purity. The manuscript underline their former developed deep learning framework, called NeuSomatic, was best somatic mutation caller. Somatic mutation detection is very import for cancer/disease study. The Sanger, DKFZ, Broad institute and other group did a broad study on how different somatic mutation caller performed in the ICGC and the TCGA's whole genome sequencing data (The ICGC/TCGA Pan-Cancer Analysis of Whole Genomes Consortium, Nature 2020; Alioto, et al. Nat Commun 2015; Pei et al, Briefings in Bioinformatics 2021). In title and abstract, the author emphasis that "Deep Learning Models" was used for robust somatic mutation detection, but only one deep learning method, NeuSomatic, was used in their study.

Another small problems, the author said the NeuSomatic was first deep learning framework for somatic mutation caller, but as my poor knowledge, DeepVariant (Poplin et al., Nat. Biotech 2018) was the first one, which was showed in the NeuSomatic paper.

*Thank you for your time in providing comments on our study and reference publications regarding other benchmarking studies. However, we would like to point out that DeepVariant, the other tool using deep learning models, is for germline variant calling. Per the note appearing on the DeepVariant website, “For somatic data or any other samples where the genotypes go beyond two copies of DNA, DeepVariant will not work out of the box because the only genotypes supported are hom-alt, het, and hom-ref.”; it suggested that DeepVariant is not suitable for detection of somatic mutations, genetic alterations acquired during the process of tumorigenesis. On the other hand, NeuSomatic is designed to identify cancer mutations in comparison to a matched normal sample.*

*In the study of ICGC/TCGA Pan-Cancer, 2020, the authors performed mutation profiling of 2658 cancer genomes across 38 tumor types with modest reads coverage on each sample (40X -60X). However, no reference call set was provided for any samples or tumor types. In addition, raw data were deposited to an access-controlled repository. Therefore, this data set is not suitable for a benchmarking study.*

*In the study by Alioto, et al., 2015, two tumor-normal pairs (chronic lymphocytic leukaemia (CLL) and medulloblastoma (MB)) were profiled with 200X-300X WGS and validated by target capture. However, its reference call set was not well-defined, i.e. 5 tiers of a total of 1,620 mutations. As stated by the authors, “This approach was limited by technical issues inherent to target capture and sequencing on these other platforms, which led to a low rate of independently validated mutations. Moreover, the real false-negative (FN) rates were underestimated because of the limited coverage provided to participants.” Therefore, we don’t think the data set from Alioto et. al. can provide an accurate assessment of the performance of mutation callers.*

*In the study by Pei et al., 2021, the authors performed benchmarking on germline and somatic mutation callers, however, data used for somatic mutation benchmarking were simulated by mixing reads from HG001 into HG002 and using the difference of germline variants between HG001 and HG002 as the “truth set” for performance evaluation. As is well-known, germline variants cannot fully resemble the characteristics of mutations acquired through tumorigenesis; the effects of other factors, such as aneuploidy of chromosome and copy number variations, were completely overlooked by this study.*

*In contrast, the dataset used in our study for model training and benchmarking has been well-characterized by the SEQC2 consortium. The “truth set” was defined with a comprehensive process yielding a high validation rate (>99.9%) (Fang et. al, Nature Biotechnology, 2021) and used in benchmarking the performance of WGS and WES studies (Xiao et.al, Nature Biotechnology, 2021). Therefore, we believe our study has important advantages over previous benchmarking studies. Moreover, its conclusions have been validated by two independent data sets (Additional file 1 Fig. S22).*

In general, Sahraeian et al.'s work did a comprehensive comparison on somatic mutation discovery in cancer genomics data, but some similar work already published. My suggestion is the manuscript not suit for publishing in Genome Biology.

My suggestion is the manuscript with following major revision:

1. Added DeepVariant and VarScan2 to the comparison.

*As we pointed out above, DeepVariant was designed for the detection of germline variant calling. DeepVariant and NeuSomatic are tools to identify genetic variants with different biological meanings. Therefore, we don’t think it is appropriate to include DeepVariant in our comparison study.*

*In addition, poor performance of VarScan2 has been demonstrated in multiple previous publications (Pei et al., Briefings in Bioinformatics, 2021, Alioto, et al., Nat Commun., 2015, Fang, L. T. et al., Genome Biol. 16, 197, 2015).*

*In our study, we have already included nine of the most recent and well-recognized tools. We believe tools selected for benchmarking in this study represented a wide variety of algorithms. Therefore, we don’t think it is appropriate to add DeepVariant and VarScan2 to the comparison.*

2. Test the power of deep learning model in ICGC/TCGA's WGS/WES data at least three cancer type.

*As mentioned above, ICGC/TCGA Pan-Cancer only performed mutation profiling of 2658 cancer genomes across 38 tumor types but did not provide reference call sets for any samples or tumor types. As a result, we cannot use ICGC/TCGA's WGS/WES data directly for a benchmarking study. However, Cooke DP, et. al (Nature Biotechnology 39, p885–892, 2021) has used mutations derived from pancreatic cancer and breast cancer to simulate two data sets for benchmarking.*

*The fastq files generated by Cooke DP et. al. are available in NCBI/SRA under project PRJNA694520, and its truth set is available via following link: https://doi.org/10.6084/m9.figshare.13902212*

*We have therefore included these two data sets in our study and repeated performance evaluation of NeuSomatic models along with eight other mutation callers. Similar results for performance metrics were achieved (Additional file 1 Fig. S22).*

3. Time the computational time and how many computer resource costed by each method.

*Thank you for the suggestion; we have benchmarked the CPU time required to run each tool. Results were presented in Additional file 2: Table S10.*

4. Literature review was limited in SEQC-II project, other similar work should be cited, both genomic data and method.

*Thank you for the suggestion. We expanded our citations to include the following references for data, benchmarking, and methods studies.*

*1. The ICGC/TCGA Pan-Cancer Analysis of Whole Genomes Consortium. Pan-cancer analysis of whole genomes. Nature 2020;578:82–93.*

*2. Alioto T, et al. A comprehensive assessment of somatic mutation detection in cancer using whole-genome sequencing. Nat Commun. 2015;6:10001.*

*3. Pei S, Liu T, Ren X, Li W, Chen C, Xie Z. Benchmarking variant callers in next-generation and third-generation sequencing analysis. Briefings in Bioinformatics. 2021; 22:3, bbaa148.*

*4. Zook JM, et al. Integrating human sequence data sets provides a resource of benchmark SNP and indel genotype calls. Nat Biotechnol. 2014;32:246–251.*

Reviewer #2: This manuscript assesses the accuracy of several computational methods, including a deep-learning approach, for calling somatic mutations from short-read sequencing data. A number of different training strategies are evaluated and accuracy is assessed via a set of higher confidence mutations from a held out region of HCC1395. Using the SEQC-II data set, performance is compared across a several of different sequencing settings (WGS, WES, fresh, FFPE, etc.)

Overall, this is a comprehensive study that describes training-based improvements to a previously developed DL method, resulting in higher accuracy. It is likely to be of interest to those working in the important field of somatic mutation detection and to those developing predictive methods for related problems.

*We really appreciate your time in reviewing this manuscript and providing positive feedbacks on its value to readers, as well as comments for its improvement. The goal of this study is not only to demonstrate the performance of NeuSomatic in the detection of cancer mutations, but also to provide recommendations for the best strategy for building a deep learning model to achieve robust performance. We concluded that it is critical to include real world data to build a deep learning model; however, a more reliable approach is actually to use a combination of real and simulated data. We think this finding will be of interest to the community developing tools for cancer mutation detection and provide some guidance in future development of predictive methods.*

Comments:

1. The text is vague and imprecise in several places to such a degree that it becomes difficult to determine what was done. For example:

a- the definition of TP and FP, FN, etc. should be more precise:must the indel be predicted exactly? Presumably the SNV base must also be called correctly. These should be stated directly.

*Thank you for pointing it out. We have added TP, FP, and FN to the list of abbreviations. Note that we don’t use these abbreviations explicitly but do refer to the truth sets described extensively in the study by Fang et Al. (Nature Biotechnology, 2021).*

*We have also added a note in the methods section, stating that “For both SNVs and INDELs, a variant call is only assumed to be correct if it exactly matches a ground truth variant”.*

b- too terse to be understandable: line 151 "Through tumor-normal titration of WGS samples,'

*Thank you for pointing it out. We have rephrased the sentence and clarified how tumor-normal mixtures were made.*

c- the experimental set up described in the "Effect of FFPE processing" section is not clear. (line 181-187)

*We have revised this section hoping that it is now clear in explaining how FFPE samples were generated.*

d- line 241: "private false negative calls" is not defined.

*Thank you for pointing it out. We have defined it in the Methods section.*

e- Section "Training data sets and models", the word "identified" is used repeatedly in this section, but it is not clear what that means. How were these candidate mutations identified? It's particularly not clear since in some cases these data sets were created by synthetically implanting mutations.

*Thank you for your comment. Here, we meant the set of candidate mutations which are detected by scanning the alignment. We reworded each instance to provide explicit clarification.*

2. The main methodological innovation is the construction and evaluation of various strategies for training the NeuSomatic DL model. While the approaches are reasonable, the methodological innovation is moderate. More critically, it's not clear to what extent these strategies generalize to other DL or ML-based approaches. Are there other DL or ML-based approaches to which the same training sets could be applied to determine how specific to NeuSomatic the approaches are?

*Thank you so much for the suggestion. We completely agreed with your comment and tested the approach with Octopus, a recently published somatic mutation detection tool with a machine learning algorithm (Cooke DP et. al, Nature Biotechnology 39, p885–892, 2021).*

*Basically, we applied four strategies, DREAM3, SEQC-WGS-Spike, SEQC-WGS-GT-50, and SEQC-WGS-GT50-SpikeWGS10, to train Octopus prediction models. Models derived from the SEQC-WGS-GT50-SpikeWGS10 data set always had the best performance on a variety of data sets (Additional file 1 Fig. S7-11). These results were consistent with results from the NeuSomatic deep learning model building, suggesting that the strategy can be generalized to other DL- or ML-based approaches.*

3. Links to source code for construction of the training data, and the ability to run the NeuSomatic predictor to reproduce results is needed. Ideally, source code to reproduce all the experiments in the paper should be provided.

*All related source codes, models, and data sets have been deposited to a NCBI ftp site that is designated to SEQC2 consortium studies (ftp://ftp-trace.ncbi.nlm.nih.gov/ReferenceSamples/seqc/Somatic_Mutation_WG).*

**Second round of review**

**Reviewer 2**

The authors have adequately addressed my previous comments.
